# Combined single-cell transcriptome and Mendelian randomization to identify and validate prognostic genes associated with endoplasmic reticulum stress and butyrate metabolism in lung adenocarcinoma

**DOI:** 10.3389/fgene.2026.1781852

**Published:** 2026-03-26

**Authors:** Jiaxin Li, Fangling Shen, Jianhua Zha, Molin Zhou, Ningbo Yi, Kang Li, Yongxin Li

**Affiliations:** 1 Department of Pharmacy, Jiangxi University of Chinese Medicine, Nanchang, China; 2 College of Health and Hygiene, Nanchang Vocational University, Nanchang, China; 3 Department of Thoracic Surgery, The First Affiliated Hospital of Nanchang University, Nanchang, China; 4 Jiangxi Hospital of China-Japan Friendship Hospital, National Regional Center for Respiratory Medicine Nanchang, Nanchang, China; 5 School of Clinical Medicine, Jiangxi University of Chinese Medicine, Nanchang, China; 6 College of Chinese Medicine and College of Life Sciences, Jiangxi University of Chinese Medicine, Nanchang, China

**Keywords:** butyrate metabolism, lung adenocarcinoma, macrophagedifferentiation, single-cell transcriptomics, TXNRD1, VDAC1

## Abstract

**Background:**

Lung adenocarcinoma (LUAD) is a prevalent and aggressive subtype of lung cancer, with a 5-year survival rate below 20% due to late-stage diagnosis and drug resistance. Endoplasmic reticulum stress (ERS) and butyrate metabolism (BM) play critical roles in tumor progression, but their co-regulatory features in LUAD remain unclear.

**Methods:**

This study integrated single-cell transcriptome analysis and Mendelian randomization (MR) to identify prognostic genes associated with ERS and BM in LUAD. Public datasets were analyzed using weighted gene co-expression network analysis, differential expression analysis, and MR. A risk model and nomogram were constructed, and immune microenvironment, gene set enrichment, and single-cell analyses were performed to validate findings. Moreover, the expression of prognostic genes was validated in different Non-small cell lung cancer (NSCLC) cell lines through reverse transcription quantitative polymerase chain reaction (RT-qPCR).

**Results:**

Seven prognostic genes (*VDAC1*, *TXNRD1*, *GDF15*, *TRIB3*, *LPL*, *KCNQ1*, *PKP2*) were identified, RT-qPCR assays confirmed that these genes exhibited significant expression differences in different NSCLC cell lines. The risk model demonstrated that low-risk patients had significantly better survival outcomes. The nomogram exhibited strong predictive accuracy for 1-, 3-, and 5-year survival. Enriched pathways in high-risk patients included olfactory transduction, while low-risk patients showed enrichment in ribosome and complement-coagulation cascades. Immune profiling revealed 13 differentially abundant immune cell types, including M1 macrophages. Single-cell analysis identified macrophages as key players in LUAD. Notably, *VDAC1*, *TXNRD1*, and *LPL* were highly expressed during early macrophage differentiation.

**Conclusion:**

This study identifies seven ERS- and BM-related prognostic genes and highlights macrophages as pivotal in LUAD progression, the expression differences of candidate genes were verified by RT-qPCR assay. These findings provide novel insights into LUAD diagnosis, prognosis, and potential therapeutic targets, offering a foundation for precision medicine strategies. Further validation in clinical cohorts and functional studies is warranted to translate these discoveries into clinical applications.

## 1 Introduction

Lung adenocarcinoma (LUAD) accounts for 40%–50% of global lung cancer cases (Wei et al., 2023; Al-Dherasi et al., 2021). Given the insidious early manifestations, roughly 60% of patients exhibit distant metastases upon diagnosis, contributing to a 5-year survival rate that remains below 20% (Denisenko et al., 2018). This disease has over two million new cases annually. High-risk groups (HRG) include smokers, individuals long-term exposed to air pollution, and those with gene mutations such as EGFR and KRAS (Liang et al., 2024; Chen and Cubillos-Ruiz, 2021). Its pathogenesis involves mutations in driver genes (EGFR/ALK/ROS1, etc.), epigenetic dysregulation, and changes in the tumor microenvironment. Recent studies have further revealed that nuclear translocation of the insulin receptor (IR) drives ERK signaling to promote LUAD growth (Ren et al., 2025), while overexpression of RRM2 correlates with cell cycle dysregulation, aggressive phenotypes, and poor prognosis (Wang et al., 2025). Moreover, DNA methylation-based signatures—such as an 8-CpG prognostic model—have demonstrated robust predictive value for overall survival, underscoring the clinical utility of multi-omics integration (Li et al., 2024). Although surgery combined with targeted therapy (such as osimertinib) or immunotherapy (PD-1/PD-L1 inhibitors) has been prevalently used, the problem of drug resistance is prominent, with a patient response rate of only 20%–30% (Chen et al., 2023a). Therefore, there is an immediate need to explore novel biomarkers and analyze tumor heterogeneity to promote precision medicine for LUAD.

Endoplasmic reticulum stress (ERS) is an adaptive mechanism by which cells maintain homeostasis through the unfolded protein response (UPR), playing a critical role in the regulation of cell survival and apoptosis (Wang and Zhang, 2018). Butyrate metabolism (BM), as a product of dietary fiber metabolism by gut microbiota, not only provides energy for the intestine but also exhibits anticancer potential by regulating inflammation and immune responses (Zhang et al., 2021; Liu Y. et al., 2024). Studies have shown that both ERS and abnormal BM can affect the tumor microenvironment: the former promotes tumor immune escape, while the latter inhibits tumor development by regulating the gut microbiota-immunity axis (Liu et al., 2018; Cheng et al., 2023). In LUAD, ERS enhances the invasiveness and chemoresistance of tumor cells and induces an immunosuppressive microenvironment by activating the IRE1α/XBP1 signaling pathway (Bischoff et al., 2021). Butyrate may downregulate pro-cancer gene expression by inhibiting HDAC activity or enhance antitumor immune responses by regulating Treg cell function (Liu Y. et al., 2024). However, the mechanism of their synergistic action in LUAD remains unclear. Elucidating the interaction between ERS and BM is able to offer new strategies for targeted therapy of LUAD. Accumulating evidence suggests a dynamic and context-dependent interplay between butyrate metabolism and endoplasmic reticulum stress, particularly within the tumor microenvironment. Butyrate, a short-chain fatty acid produced by gut microbiota and also utilized by host cells as an energy substrate or signaling molecule, functions not only as a histone deacetylase (HDAC) inhibitor but also as a modulator of cellular stress responses. Notably, butyrate has been shown to directly influence ERS signaling pathways. In colorectal cancer models, butyrate treatment upregulated key ERS markers—including GRP78/BiP, CHOP, and IRE1α—and induced autophagy, suggesting that butyrate can potentiate the unfolded protein response (UPR) under certain metabolic conditions (Wei et al., 2023; Al-Dherasi et al., 2021). Conversely, in inflammatory contexts such as colitis-associated cancer, butyrate was reported to attenuate ERS by suppressing the STING–ER stress axis, thereby exerting anti-inflammatory and cytoprotective effects (Denisenko et al., 2018). This dual role underscores the context-specific nature of butyrate–ERS crosstalk.

Mechanistically, HDAC inhibition by butyrate alters the acetylation status and subcellular localization of ER chaperones like GRP78, thereby modulating UPR activation and cell fate decisions during stress (Liang et al., 2024; Chen and Cubillos-Ruiz, 2021). Emerging evidence also highlights the role of non-coding RNA networks in fine-tuning this crosstalk; for instance, the MALAT1/miR-140-5p axis has been shown to regulate Nrf2-mediated antioxidant responses under cellular stress (Qin and Xu, 2024), suggesting that analogous lncRNA–miRNA circuits may operate in LUAD to coordinate ERS and metabolic adaptation. Given that both ERS and butyrate metabolism shape immune cell function (e.g., macrophage polarization) and stromal interactions in the tumor niche (Chen et al., 2023a), their convergence may create a unique “stress-metabolic” regulatory node in LUAD progression.

Therefore, integrating ERS and butyrate metabolism is not merely additive but biologically synergistic: their intersection likely harbors master regulators that coordinate proteostasis, immunometabolism, and therapeutic resistance in LUAD—features that cannot be captured by studying either pathway in isolation.

Single-cell RNA sequencing (scRNA-seq) can resolve the heterogeneity of gene expression with high resolution, revealing cell subpopulations, developmental trajectories, and intercellular interaction networks, which is crucial for understanding the dynamics of the tumor microenvironment (TME) (Cheng et al., 2023). MR uses genetic variations as instrumental variables to identify causative associations while controlling for confounding bias, based on three core assumptions: association, independence, and exclusivity (Bischoff et al., 2021; Wang et al., 2022). In LUAD research, scRNA-seq technology has identified fibroblast subpopulations that promote tumor growth and dysfunctional T-cell populations associated with immunotherapy resistance (Bowden and Holmes, 2019). MR studies have confirmed the causal associations between environmental factors (smoking, PM2.5) and LUAD risk, as well as the impact of metabolic-related genetic variations (such as FTO) on disease progression (Li et al., 2023). Although these two methods have been independently applied to LUAD research, their combined use for exploring the ERS and BM regulatory networks remains unexplored. This strategy can simultaneously identify cell type-specific gene expression patterns and causal pathways.

This study systematically explores the molecular regulatory networks and clinical values of ERS and BM in LUAD based on transcriptome data of LUAD from public databases, integrating MR and multi-dimensional bioinformatics analyses. Causal genes significantly associated with ERS/BM were screened via MR to construct a prognostic risk score model and a clinical nomogram model. Combined with tumor immune microenvironment analysis, the study reveals their potential roles in regulating immune cell infiltration and checkpoint expression. Meanwhile, single-cell sequencing was utilized to identify key cell subsets with high expression of ERS/BM-related genes in the tumor microenvironment. Through cell communication network and pseudotime trajectory analyses, the dynamic regulatory mechanisms of these genes in tumor metastasis and drug resistance evolution were elucidated. For the first time, this study integrates MR causal inference with single-cell multi-omics, providing an evidence chain at the cellular level for the pathological mechanisms of ERS/BM in LUAD. By constructing high-precision prediction models and discovering potential therapeutic targets, it offers a new framework and data support for early diagnosis, stratified treatment, and the development of immune-metabolic combination therapies.

## 2 Materials and methods

### 2.1 Data collection

The TCGA-LUAD dataset was acquired from the Cancer Genome Atlas (TCGA) on 11 December 2024. This dataset included 515 LUAD tumor tissue samples with survival information and 59 adjacent normal lung tissue samples. The TCGA-LUAD dataset also contained patients’ clinical data, such as age, overall survival (OS) time, gender, and survival status. Meanwhile, the GSE31210 (GPL570) and GSE131907 (GPL16791) datasets were gained from the Gene Expression Omnibus (GEO). In GSE31210 dataset, 226 LUAD tumor tissue samples were gained for analysis. The GSE116959 (GPL17077) dataset included 57 LUAD tumor tissue samples and 11 control tissue samples, and this dataset was used for the verification of gene expression levels. The GSE131907 dataset contained 11 LUAD tumor tissue samples and 11 normal lung tissue samples. In addition, 1,350 ERS-related genes (ERS-RGs) (Deng et al., 2023) and 358 butyrate-metabolism-related genes (BM-RGs) (Supplementary Table S1) were gained from the literature.

### 2.2 Weighted gene co-expression network analysis (WGCNA)

In this study, based on 358 BM-RGs, univariate Cox analysis and proportional risk test were performed with the “survival” package to screen BM-RGs with prognostic value, and then a single-sample gene set enrichment analysis was performed with the gene set variation analysis (GSVA) package on the TCGA-LUAD cohort to compute the patients’ BM-RG scores and group them according to the optimal cutoff value (Hänzelmann et al., 2013). Kaplan-Meier survival analysis was performed with the “survminer” package (Shi et al., 2023). Subsequently, a weighted gene co-expression network was constructed using the “WGCNA” package (Langfelder and Horvath, 2008), outliers were excluded by hierarchical clustering, soft thresholds were determined based on the scale-free fit index and average connectivity, and gene modules (≥500 genes per module, with a merging threshold of 0.3) were divided using a hybrid dynamic tree-cutting algorithm. Module correlations were calculated using BM-RGs scores as phenotypes, and genes within the strongest positively and negatively correlated modules were defined as WGCNA-BM-RGs.

### 2.3 Identification and enrichment analysis of candidate genes

In the TCGA-LUAD dataset, DEGs (|log_2_ fold change (FC)| > 0.5 and P < 0.05) between tumor and normal lung tissues were screened by the DESeq2 package (v 1.38.0) (Love et al., 2014), the core was visualized using ggplot2 (v 3.3.6) (Gustavsson et al., 2022) and ComplexHeatmap (v 2.14.0) (Gu, 2022) differential genes, and the intersection of DEGs, ERS-RGs, and WGCNA-BM-RGs were identified as candidate genes by VennDiagram (v 1.7.3) (Chen and Boutros, 2011). Subsequently, gene ontology (GO) (biological processes (BP)/cellular components (CC)/molecular functions (MF)) and kyoto encyclopedia of genes and genomes (KEGG) pathway enrichment analyses (P < 0.05) were performed using the clusterProfiler (v 4.6.2) (Wu et al., 2021) package, and the top 5 GO entries and the top 10 KEGG pathways were visualized by ggplot2 and GOplot (Walter et al., 2015) respectively, in order to resolve the biological functions of the candidate genes and signaling networks.

### 2.4 The construction of protein-protein interaction (PPI) network

The PPI network of candidate genes was built using the Search Tool for the Retrieval of Interacting Genes/Proteins (STRING) with a confidence level higher than 0.4. The findings were then illustrated using Cytoscape software (v 3.10.2) (Doncheva et al., 2019).

### 2.5 Mendelian randomization (MR) analysis

To ensure robust causal inference between gene expression and lung adenocarcinoma (LUAD) risk, we conducted a two-sample MR analysis using expression quantitative trait loci (eQTLs) as instrumental variables (IVs). To minimize the risk of horizontal pleiotropy, which is substantially higher for trans-eQTLs, we strictly limited our analysis to cis-eQTLs. Specifically, for each of the 86 candidate genes, we extracted SNPs from the Genotype-Tissue Expression (GTEx) project (v8, lung tissue) that met two criteria: (1) a genome-wide significant association with the gene’s expression (P < 5 × 10^−6^), and (2) physical location within ±1 megabase (Mb) of the gene’s transcription start site (TSS).

Crucially, to address the potential for sample overlap which can inflate Type I error rates, we performed a thorough verification of the independence between the exposure and outcome datasets. The GTEx project (v8) comprises post-mortem samples from donors who were not participants in large-scale disease-focused biobanks like UK Biobank. In contrast, the ILCCO/TRICL GWAS meta-analysis is a collaborative effort that aggregates data from numerous independent case-control studies and cohort studies worldwide, none of which contributed samples to the GTEx v8 lung tissue dataset. Based on the publicly available cohort descriptions and sample provenance information, we confirm that the eQTL data (exposure) and the LUAD GWAS data (outcome) originate from entirely non-overlapping sets of individuals. This strict separation of samples is a fundamental safeguard against bias arising from sample overlap in our MR framework.

Candidate SNPs were then pruned for linkage disequilibrium (LD) using the clumping function in the TwoSampleMR R package (v0.5.6), with a 10 kb window and an LD threshold of *r* ^2^ < 0.001, based on the 1000 Genomes Phase 3 European (EUR) reference panel. This stringent *r* ^2^ criterion minimizes residual correlation among retained SNPs and reduces bias from LD contamination (Al-Dherasi et al., 2021).

To guard against weak instrument bias, we calculated the F-statistic for each potential IV. The F-statistic was computed as

$$F=\frac{R^{2}\left(N‐K‐1\right)}{\left(1‐R^{2}\right)K}$$

where N is the sample size of the exposure dataset (GTEx lung tissue, N ≈ 495), K is the number of IVs for a given gene (K = 1 for individual SNP assessment), and *R* ^2^ is the proportion of variance in gene expression explained by the SNP. Only SNPs with an F-statistic >10 were retained as valid IVs, ensuring sufficient instrument strength. Additionally, to ensure stable causal estimation, we required at least three independent SNPs per gene; genes failing this criterion were excluded.

Potential horizontal pleiotropy was addressed by screening all SNPs against the PhenoScanner v2 database; SNPs associated (P < 5 × 10^−8^) with known confounders (e.g., smoking, BMI, or other cancers) were excluded (Liang et al., 2024). Allele harmonization between exposure and outcome datasets was performed using the harmonise_data function. The primary causal estimate was derived using the inverse-variance weighted (IVW) method, complemented by sensitivity analyses including MR-Egger regression, weighted median, and MR-PRESSO to assess pleiotropy and robustness. Heterogeneity was evaluated via Cochran’s Q test (P > 0.05 indicating no significant heterogeneity), and the leave-one-out analysis assessed result stability. Directionality was confirmed using the Steiger filtering test (Steiger_P < 0.05), ensuring that genetic variants explained more variance in the exposure than in the outcome. While the ideal validation of SNP-gene associations would involve an independent cohort with matched tissue and ancestry, sourcing such a dataset for all 86 candidate genes presents significant practical challenges. Publicly available eQTL resources often differ in tissue processing protocols, genotyping platforms, and ancestral composition, and direct cross-dataset comparisons can introduce substantial heterogeneity and bias.

Therefore, to ensure the highest possible reliability of our instrumental variables without introducing cross-cohort artifacts, we implemented a rigorous, multi-layered quality control pipeline within our primary eQTL source (GTEx v8, lung tissue). This pipeline included: (1) stringent statistical significance (P < 5 × 10^−6^); (2) strict physical proximity (cis-eQTLs within ±1 Mb of the TSS); (3) robust instrument strength (F-statistic >10 for all SNPs); (4) LD clumping to ensure independence (*r* ^2^ < 0.001); (5) comprehensive screening for horizontal pleiotropy using the PhenoScanner database; and (6) confirmation of causal directionality via the Steiger filtering test. By adhering to these stringent criteria, we have maximized the confidence that our selected SNPs are valid, strong, and specific instruments for their respective target genes, thereby upholding the core assumptions of Mendelian randomization. Only genes passing all quality control steps were retained as candidate prognostic genes.

### 2.6 Identification of prognostic genes and development and validation of predictive risk models

In the disease samples of TCGA-LUAD, by means of the candidate prognostic genes, univariate Cox analysis (P < 0.05) and the PH test (P > 0.05) were carried out using “survival” package (v 3.5-3) (Liu T. T. et al., 2021). Genes that were ascertained as either risk factors or protective factors in both the univariate Cox analysis and MR analysis were selected as prognostic genes for this study. In accordance with the prognostic genes, a random survival forest model was constructed using “randomForestSRC” package (v 3.2.2) (Zhao et al., 2023). For each individual patient in TCGA-LUAD, the corresponding risk score was computed. In accordance with prime cut-off value of risk scores, disease samples in TCGA-LUAD were sorted into a HRG and a LRG. Then, survival analysis was carried out using “survminer” package (v 0.4.9), and the K-M curve was plotted (P < 0.05). Receiver Operating Characteristic (ROC) analysis was executed applying “survivalROC” package (v 1 0 3 1) (Chen et al., 2023b). ROC curves were generated for time periods of 1-, 3-, and 5-year, and the corresponding area under curve (AUC) values were calculated (AUC > 0.7). Furthermore, the expression disparities of prognostic genes within HRG and LRG were discriminated. The same method was employed to validate the risk model in GSE31210.

### 2.7 Expression of prognostic genes and their relationship to survival in LUAD patients

The Wilcoxon test was availed to compare expression differences of prognostic genes within disease samples and control samples in TCGA-LUAD. Furthermore, the Human Protein Atlas (HPA) database was utilized to explore the differences in protein contents of prognostic genes within disease samples and control samples. Meanwhile, to understand the survival relationship within the prognostic genes and LUAD patients, in TCGA-LUAD, LUAD were sorted into a high-expression group (HEG) and a low-expression group (LEG) of prognostic genes. The “survival” package (v 3.5-3) was used to conduct K-M survival analysis to evaluate the distinction in OS time within HEG and LEG (P < 0.05).

### 2.8 Development and assessment of a nomogram model

In TCGA-LUAD samples, we conducted univariate Cox regression (HR ≠ 1, P < 0.05) and PH testing (P > 0.05) on risk score, demographic variables, and TNM staging to identify potential prognostic factors. To ensure the validity of the subsequent multivariate Cox model, we verified the proportional hazards (PH) assumption for all candidate variables using Schoenfeld residuals tests. Variables that met the PH assumption (P > 0.05) were then included in the multivariate analysis. Factors meeting significance thresholds were further analyzed via multivariate Cox regression to determine independent prognostic indicators. Based on these factors, we constructed a nomogram using the rms package to predict 1-, 3-, and 5-year OS in LUAD patients (Liu T. T. et al., 2021). Model performance was evaluated via calibration curves (using regplot), with slopes approaching 1 indicating optimal predictive accuracy (Zhu et al., 2024). Meanwhile, ROC analysis was executed and the AUC value was computed (AUC > 0.7) using “survivalROC” package. Moreover, within the TCGA-LUAD cohort, patients with LUAD were categorized into distinct clinical subgroups based on their varying clinical features. Disparities in risk scores within diverse clinical subgroups were discriminated using the Wilcoxon test (P < 0.05), and visualization was carried out using “ggplot2” package.

### 2.9 Gene set enrichment analysis (GSEA)

To explore the signally enriched biological pathways in HRG and LRG, in disease samples of TCGA-LUAD, differential analysis within the HRG and LRG was carried out using “DEseq2” package. The log_2_FC values were computed and sorted in descending order based on these values. Subsequently, GSEA was executed using “clusterProfiler” package (P < 0.05, |Normalized Enrichment Score (NES)| > 1). The reference gene set h.all.v2023.2.Hs.symbols.gmt was gained from the MSigDB database for application in this study. Moreover, to explore biological pathways in which the prognostic genes were involved, in TCGA-LUAD, the gene set c2.cp.kegg.v7.4.symbols.gmt was leveraged as the reference gene set. The “psych” package (v 2 2 9) (Robles-Jimenez et al., 2021) was availed to calculate the spearman connection coefficients within prognostic genes and other genes (|cor| > 0.3, P < 0.05), and the genes were sorted in descending order In accordance with the connection coefficients. Subsequently, GSEA analysis was carried out via “clusterProfiler” package (|NES| > 1, P < 0.05). The “enrichplot” package (v 1.18.0) (Zhang et al., 2019) was then harnessed to display the top 5 pathways sorted in descending order of significance.

### 2.10 Immune microenvironment and gene mutation analysis

In the disease samples of TCGA-LUAD, CIBERSORT algorithm was harnessed to assess infiltration proportions of 22 types of immune cells in HRG and LRG (Chen et al., 2018). Subsequently, the Wilcoxon test was utilized to show infiltration differences of 22 types of immune cells within HRG and LRG. Immune cells exhibiting noteworthy differences (P < 0.05) were pinpointed, and “ggplot2” package was applied to generate box plots for visualizing the findings. Subsequently, “psych” package was utilized to examine spearman connections within differential immune cells and within differential immune cells and prognostic genes (|cor| > 0.3, P < 0.05). In TCGA-LUAD, to explore potential clinical efficacy of immunotherapy in HRG and LRG, “ESTIMATE” algorithm was harnessed to estimate immune, stromal, and ESTIMATE score. The Wilcoxon test was then utilized to examine differences in immune score, stromal score, ESTIMATE score, and immune checkpoints within patients in HRG and LRG (P < 0.05). The “MAfTools” package (v 2.14.0) (Mayakonda et al., 2018) was utilized to draw a waterfall plot to display the top 20 genes with the highest mutation frequencies. Meanwhile, “MAfTools” package was utilized to ascertain tumor mutation burden (TMB) of HRG and LRG. The Wilcoxon test was employed to examine difference in TMB within HRG and LRG (P < 0.05).

### 2.11 Construction of molecular regulatory networks

In MicroCosm database and miRanda database, microRNAs (miRNAs) targeting the prognostic genes were predicted. The union of the prediction results from the two datasets was taken to derive the final miRNAs. Meanwhile, the upstream long non-coding RNAs (lncRNAs) targeting the miRNAs (clipExpNum > 4) were gained from starBase database (https://starbase.sysu.edu.cn/). Additionally, transcription factors (TFs) targeting the prognostic genes were predicted through JASPAR database (https://jaspar.genereg.net/). Finally, the lncRNA-miRNA-mRNA network and TFs-mRNA network were constructed using Cytoscape software.

### 2.12 Drug prediction and molecular docking

Targeted drugs for the prognostic genes were identified using the DSigDB plugin of the Enrichr database (https://maayanlab.cloud/Enrichr/), and visualized with Cytoscape software. Subsequently, the three-dimensional structures of the drugs with the highest scores for each prognostic gene were retrieved and downloaded from PubChem database (https://pubchem.ncbi.nlm.nih.gov/). Meanwhile, three-dimensional structures of the prognostic genes were retrieved and downloaded from UniProt database (https://www.uniprot.org/) and AlphaFold database (https://alphafold.ebi.ac.uk/entry/). Molecular docking was carried out using online website cb-dock (https://cadd.labshare.cn/cb-dock/php/blinddock.php). Generally, a binding energy ≤ −5.0 kcal/mol was considered to indicate a tight binding relationship, and the smaller binding energy, the better binding ability.

### 2.13 Single cell transcriptome analysis

In GSE131907, quality control was carried out using “Seurat” package (v 5.0.1) (Hao et al., 2021). The process entailed filtering out genes expressed in fewer than three cells. Additionally, we applied stringent quality control criteria to mitigate potential confounding effects of cellular stress on metabolic pathway inference. Specifically, cells were excluded if they exhibited: (i) fewer than 300 or more than 4,000 detected genes (nFeature_RNA); (ii) total UMI counts (nCount_RNA) below 500 or above 20,000; or (iii) mitochondrial gene expression accounting for more than 15% of total transcripts (percent_mt > 15%).

This 15% threshold was determined based on empirical evaluation of the mitochondrial read proportion distribution across all samples (Supplementary Figure S7A), ensuring effective removal of apoptotic or damaged cells while preserving biologically relevant cell states. Given that our study focuses on butyrate metabolism and endoplasmic reticulum stress—processes intimately linked to mitochondrial function—this conservative cutoff minimizes non-specific elevation of oxidative phosphorylation and stress-response signals in downstream pathway analyses, thereby enhancing the biological fidelity of metabolic program interpretation.

The “NormalizeData” function was harnessed to normalize the gene contents among different cells, with the parameter set as LogNormalize (scale.factor = 10,000). Then, the “FindVariableFeatures” function was harnessed to derive the highly variable genes (HVGs) with large differences in contents among cells based on the relationship between the mean and variance. The “LablePoints” function was utilized to display the top 10 genes with the largest variances. After the HVGs were ascertained, the “ScaleData” function in “Seurat” package (v 5.0.1) was utilized to normalize the data. The “RunPCA” function was utilized to execute PCA for dimensionality reduction on HVGs (P < 0.05). The “FindNeighbors” and “FindClusters” functions in “Seurat” package (v 5.0.1) were utilized to conduct unsupervised clustering analysis on the cells. The number of cell clusters was determined by the principal components (dims = 1:20, resolution = 0.2). Then, the cell subpopulations gained through uniform manifold approximation and projection (UMAP) feature reduction and clustering were annotated. The “FindAllMarkers” was utilized to recognize marker genes in each cell cohort in GSE131907 (logfc.threshold = 0.5, min.pct = 0.25, return.thresh = 0.01). In light of the marker genes, the gained different cell clusters were annotated to the known cell types using “singleR” package (v 2.4.1) (Mayakonda et al., 2018) and the CellMarker database (http://yikedaxue.slwshop.cn/). To validate and visually support the annotation, a DotPlot was generated using the DotPlot function in the Seurat package (v5.1.0) to simultaneously display the average expression level and the percentage of cells expressing canonical marker genes for major lineages, including epithelial cells (EPCAM, KRT8/18/19), myeloid cells (CD68, CD163, LYZ), T lymphocytes (CD3D, CD3E, CD8A), fibroblasts (COL1A1, DCN), and endothelial cells (PECAM1, VWF). Additionally, FeaturePlots were created using the FeaturePlot function to illustrate the spatial distribution of these key markers across the UMAP embedding. The “umap” package (v 0.2.10.0) (Liu et al., 2025) was utilized to draw the UMAP plot for displaying the annotated cell clusters. Subsequently, “ReactomeGSA” (https://github.com/reactome/ReactomeGSA) was utilized to explore the functional enrichment of the annotated cell types and investigate their biological pathways (P < 0.05). Meanwhile, the ratios of different cell types in disease samples and control samples were presented.

Furthermore, to assess the cell-type-specific activity of endoplasmic reticulum stress (ERS) and butyrate metabolism (BM), we calculated pathway scores for each individual cell. Using the AddModuleScore function in the Seurat package (v5.1.0), we computed an ERS score based on the expression of 1,350 reported ERS-related genes (ERS-RGs) and a BM score based on 358 butyrate-metabolism-related genes (BM-RGs). This method calculates a score by averaging the expression of the gene set of interest and subtracting the average expression of control gene sets randomly sampled from matched expression bins, thereby providing a robust, normalized measure of pathway activity at single-cell resolution. These scores were then stratified by annotated cell type to identify which lineages exhibit the highest ERS and BM activities.

To investigate the functional heterogeneity within key immune cell populations, we performed a focused analysis on macrophages. An endoplasmic reticulum stress (ERS) activity score for each macrophage was calculated using the AddModuleScore function in the Seurat package (v5.0.1). This score was derived from the average expression of 1,305 ERS-related genes (a subset of the reported 1,350 ERS-RGs that were expressed in our macrophage dataset), after subtracting the average expression of control gene sets of equal size randomly sampled from the same expression bins. Macrophages were subsequently stratified into ERS-high and ERS-low subgroups based on the median ERS score.

Differential gene expression analysis between the ERS-high and ERS-low macrophage subgroups was conducted using the Wilcoxon rank-sum test, with thresholds of |log_2_ fold change (FC)| > 1 and adjusted p-value <0.05. The resulting differentially expressed genes (DEGs) were then used as input for Gene Set Enrichment Analysis (GSEA) via the clusterProfiler package (v4.6.2). The reference gene set comprised 7,894 curated gene sets from the Molecular Signatures Database (MSigDB v7.5.1). GSEA parameters were set as follows: minGSSize = 10, maxGSSize = 500, pvalueCutoff = 1.0, and pAdjustMethod = “BH”. A gene set was considered significantly enriched if its Benjamini–Hochberg (BH) adjusted p-value was less than 0.05.

### 2.14 Identification of key cells

A bubble chart was employed to illustrate the content of prognostic genes across various cell types. The Wilcoxon test was employed to examine distinction in ratios of different cell types within disease samples and control samples. Cells with P < 0.05 were defined as differential cells. Meanwhile, the UMAP plot was utilized to display the distribution of prognostic genes in differential cells. The cells with the highest expression of the most prognostic genes were specified as key cells. The “FindMarkers” function in “Seurat” package (v 5.0.1) was utilized to conduct differential analysis on the genes contained in the key cells (P.adj < 0.05, |avg_log_2_FC| > 0.5). Then, “clusterProfiler” package was used to execute GO and KEGG analyses on differential genes. To understand the heterogeneity of the key cells in GSE131907, “FindAllMarkers” function was utilized to recluster the key cells into different sub-populations (min.pct = 0.5, test.use = “auc”, logfc.threshold = 0.25).

### 2.15 Cell communication and quasi-temporal analysis

To quantify the expression and pairing of receptors and ligands in the annotated cells of the single-cell dataset GSE131907 (cell-to-cell communication analysis was carried out with the disease as one sample and the control as the other sample), the CellChat database (http://cellchatdb.ibio-cn.org/) was referred to, and “CellChat” package (v 1.6.1) (Luo et al., 2023) was utilized to explore the intercellular interactions of the annotated cells. The “ggplot2” package was applied to portray the intercellular interactions. The “Monocle” package (v 2.30.1) (Trapnell et al., 2014) was utilized to conduct the pseudo-time analysis. The contents of different prognostic genes in the key cells were examined, and “DiffialGeneTest” function was utilized to explore the gene dynamics during the cell differentiation process. To understand the CytoTRACE scores of key cells, “CytoTRACE2” package (v 1.0.0) (Xu et al., 2024) was utilized to examine developmental trajectories of cells in GSE131907. Moreover, UMAP plots were utilized to display the CytoTRACE scores of different key cell subsets. A higher score indicated that the cells were relatively in the initial stage of the differentiation process. To visualize the expression of prognostic genes across various subsets of key cells, bubble plots were utilized.

### 2.16 Expression analysis

The expression of prognostic genes was analyzed in the TCGA-LUAD dataset and GSE116959 dataset respectively, and the Mann-Whitney U test was used to compare the differences between the tumor group and the normal group (p < 0.05).

### 2.17 Reverse transcription quantitative polymerase chain reaction (RT-qPCR)

We obtained HBE (a human bronchial epithelial cell line), PC9, A549, H292, H1299, HCC827 and H1975 (six human lung adenocarcinoma cell lines) supplied by Procell Life Science & Technology Co. Ltd. Total cellular RNA was extracted following the instructions of the manufacturer and using a TRIzol reagent (Invitrogen, United States). Subsequently, cDNA was generated through reverse transcription of RNA, employing PrimeScript RT Master Mix (Takara). For the Non-small cell lung cancer cell lines, the primer sequences used were (5′-3′): GAPDH (forward: 5′-CCT​CGT​CCC​GTA​GAC​AAA​ATG-3′, reverse: 5′- TGA​GGT​CAA​TGA​AGG​GGT​CGT-3), *GDF15* (forward: 5′- CTG​GCA​ATG​CCT​GAA​CAA​CG -3′, reverse: 5′- GGT​CGG​GAC​TTG​GTT​CTG​AG- 3′), *KCNQ1*(forward: 5′- CAC​CAA​TCT​GGG​ATC​AGT​CCT-3′, reverse: 5′- TGC​AGC​TTC​AAC​ACA​TAG​TCC​A-3′), *LPL*(forward: 5′- TGA​GGA​TGG​CAA​GCA​ACA​ACA​CAA​CC-3′, reverse: 5′- CAT​GAG​CAG​TTC​TCC​GAT​GTC​CAC-3′), *PKP2*(forward: 5′- TCC​GTG​GAA​GAA​AGG​TCC​TTG-3′, reverse: 5′- AAT​CGC​TGT​GCG​TGT​AGT​GAG-3′), *TRIB3*(forward: 5′- GTC​GAT​TTG​TCT​TCA​GCA​ACT​G-3′, reverse: 5′- CTG​AGT​ATC​TCT​GGT​CCC​ACA​T-3′), *TXNRD1* (forward: 5′- CAC​GGA​TGA​GGA​GCA​GAC​CAA​TG-3′, reverse: 5′- CAT​ACA​GCC​TCT​GAG​CCA​GCA​ATC-3′), *VDAC1*(forward: 5′- TTC​CAG​CTT​CAT​ACT​AAT​GTG​AAT​G-3′, reverse: 5′- CTA​AGC​CAA​TTA​GAC​TGG​AGT​TGT​T-3′) RBM47 (forward: 5′- CCT​ACA​ACG​CTC​TCA​TCG​GGC-3′, reverse: 5′- GCG​GAA​TAT​CCT​CCG​AGG​TAG G- 3′), synthesized by shanghai Generay Biotech. The primer sequence was obtained from Primer Bank (https://pga.mgh.harvard.edu/primerbank/index.html) and synthesized by Shanghai Sangon Biotechnology (Shanghai, China). The GAPDH gene was used as an internal reference gene. Calculation of the expression of the target gene was performed based on the 2^−ΔΔCT^ method.

### 2.18 Statistical analysis

R software (v 4.2.2) was leveraged to conduct all analyses. Group-wise differences were assessed via the Wilcoxon test, and statistical significance was defined as p < 0.05.

## 3 Results

### 3.1 The 2,582 WGCNA-BM-RGs were ascertained

**FIGURE 1:**
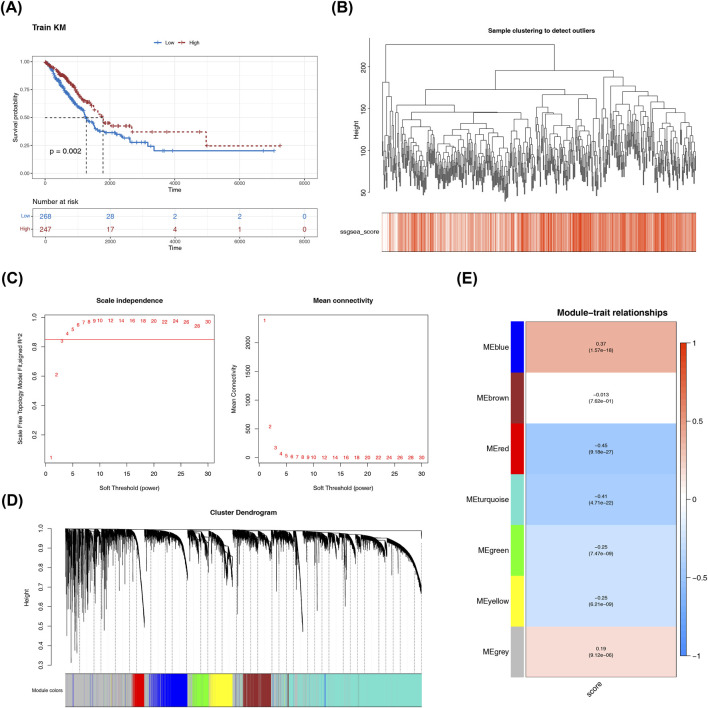
Identification of differentially expressed genes. **(A)** Enrichment scores of butyrate metabolism-related genes and Kaplan-Meier (KM) survival curves of high and low score groups. Red represented the high-score group, and blue represented the low-score group. **(B)** Clustering diagram of LUAD training set samples. Each branch represented each sample, and the ordinate was the height of hierarchical clustering. **(C)** Soft-thresholding screening. The abscissa of both the left and right figures represented the weight parameter power value; the ordinate of the left Figwas the scale-free fit index. **(D)** Dynamic tree cut of modules. **(E)** Heatmap of module-trait correlation. The abscissa was the module (each color represented a different module), and the ordinate was the M-RGs score. In each grid, the value above was the correlation coefficient R, and the value below was the significance P-value.

A total of 65 BM-RGs were associated with prognosis (Supplementary Table S2). As per the optimal cut-off value of BM-RGs score (−0.03558426), patients with LUAD from TCGA-LUAD cohort were divided into two groups: a high-score group (n = 247) and a low-score group (n = 268). The high-score group had a higher survival rate (P = 0.002) (Figure 1A). All samples in TCGA-LUAD dataset were within the expected range, with no outliers detected (Figure 1B). When the soft threshold power was selected as 4, the network exhibited an approximation of a scale-free distribution (Figure 1C). Once the soft threshold was established, co-expression network was subsequently built, which resulted in the formation of six modules (excluding the grey module that contained unclassifiable genes) (Figure 1D). The blue part had the strongest positive correlation with BM-RGs score (cor = 0.37, P = 1.57e-18), and the red part had the strongest negative connection with BM-RGs score (cor = −0.45, P = 9.18e-27) (Figure 1E). There were a total of 2,582 genes in these two key modules (blue module contains 1,953 genes, while red module includes 629 genes.), which were named WGCNA-BM-RGs.

### 3.2 Candidate genes were enriched in different pathways and had different interaction relationships

**FIGURE 2:**
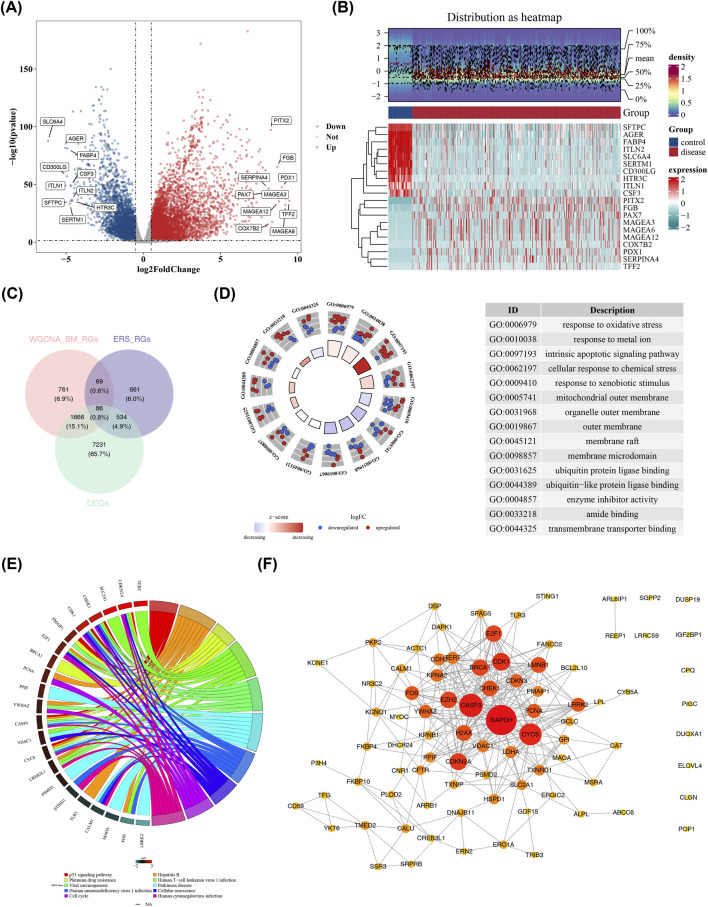
Candidate gene screening. **(A)** Volcano plot of differentially expressed genes. The X-axis represented −log_10_ (adj. p.value), and the Y-axis represented the log_2_ fold change. Each point on the plot represented a gene, with blue points indicating significantly downregulated genes, red points indicating significantly upregulated genes, and gray points indicating non-significantly different genes. **(B)** Heatmap of differentially expressed genes. The upper part showed the expression density heatmap of the 20 genes with the largest fold changes between samples, and the lower part was the expression heatmap. Each column represented a sample, and each row represented the expression level of a gene across different samples. The color of the heatmap indicated the gene expression level in the sample, with deeper colors representing higher expression levels (red for high expression and cyan for low expression). The red color in the grouping indicated the disease group, and blue indicated the control group. **(C)** Venn diagram of candidate genes. **(D)** GO enrichment analysis of candidate genes. The left half was a circular diagram of GO enrichment analysis, and the right half showed the biological processes enriched in the GO analysis. **(E)** KEGG enrichment analysis of candidate genes. The circular diagram in the figure could be divided into two parts, with the upper part showing the names of the enriched genes. **(F)** PPI network diagram of candidate genes. Circles represented candidate genes, with darker colors indicating higher degree (degree) and greater importance.

In TCGA-LUAD, there were 9,517 DEGs within disease and control samples. Among them, 6,146 DEGs were highly expressed in disease samples, and 3,371 DEGs were highly expressed in control samples (Figures 2A,B). An intersection was taken among the 9,517 DEGs, 1,350 ERGs, and 2,582 WGCNA-BM-RGs, and 86 candidate genes were gained (Figure 2C). These candidate genes were enriched in 903 GO terms, including 703 BP terms (such as response to oxidative stress and response to metal ion), 94 CC terms (such as mitochondrial outer membrane and organelle outer membrane), and 106 MF terms (such as transmembrane transporter binding and ubiquitin protein ligase binding) (Figure 2D; Supplementary Table S3). Meanwhile, they were also statistically enriched in 220 KEGG pathways (such as p53 signaling pathway and cellular senescence) (Figure 2E; Supplementary Table S4). Moreover, there were complex interaction relationships among the candidate genes, such as CYCS-PCNA and GAPDH-CHEK1 (Figure 2F). These enrichment analysis results provided crucial clues for exploring the physiological and pathological mechanisms related to LUAD, and were conducive to revealing the internal connections within the candidate genes and the disease.

### 3.3 MR analysis ascertained 29 candidate prognostic genes

**FIGURE 3:**
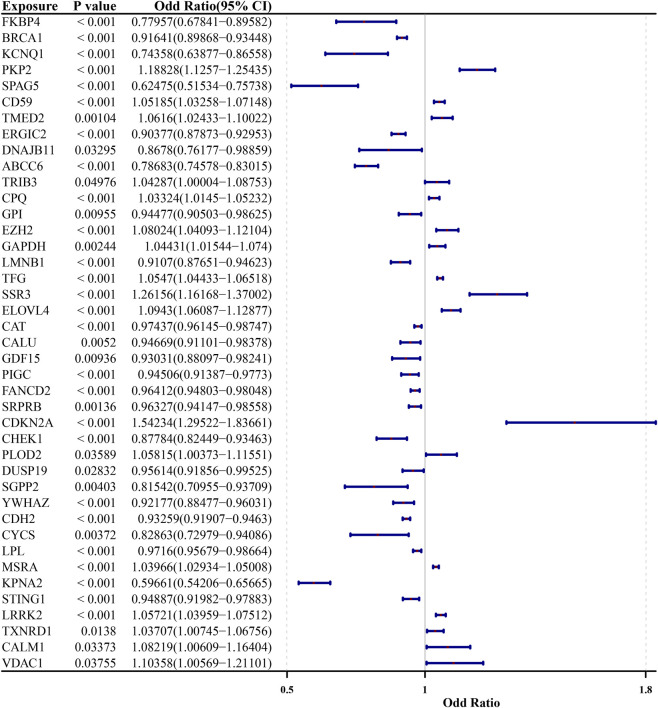
MR Randomization analysis results. MR Randomization analysis results of exposed factors (forest plot), from left to right are prognosis-related genes, corresponding p-values and hazard ratios, and their confidence intervals. Among them, the smaller the value range of the confidence interval, the greater its credibility.

Using the 86 candidate genes as exposure factors and LUAD as the event, a two-sample MR analysis was executed. It was found that there was a noteworthy connection between 41 exposure factors and LUAD (P < 0.05, OR≠1) (Figure 3; Supplementary Table S5). Among them, 24 genes were protective factors and 17 genes were risk factors. The scatter plots and forest plots also unveiled that 24 genes were protective factors and 17 genes were risk factors (Supplementary Figures S1, S2). The number of SNPs for these 41 exposure factors was symmetric on both sides of the IVW line, which was in line with Mendel’s second law (Supplementary Figure S3). Heterogeneity tests manifested that 37 out of these 41 exposure factors revealed no heterogeneity (P > 0.05) (Supplementary Table S6). The pleiotropy test unveiled that 32 genes had no pleiotropy (P > 0.05) (Supplementary Table S7). The LOO analysis manifested that none of the 41 exposure factors had severely deviated points, suggesting the validity of the results (Supplementary Figure S4). Finally, through the Steiger directionality test, 29 exposure factors without reverse causation were gained (Supplementary Table S8), which were recorded as candidate prognostic genes.

### 3.4 The risk model was constructed and validated based on 7 prognostic genes

**FIGURE 4:**
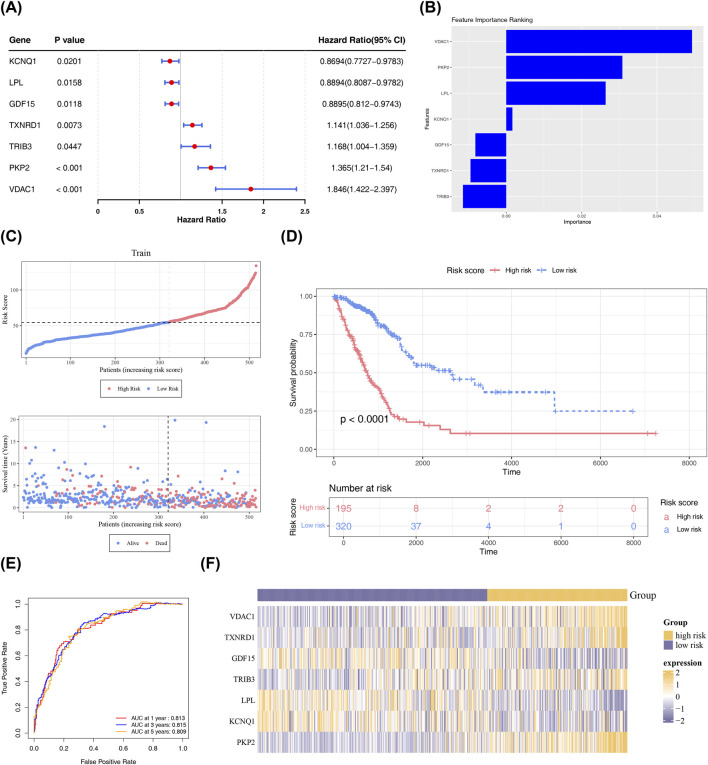
Construction of risk model. **(A)** Univariate COX forest plot. From left to right are the prognosis - related genes, the corresponding P value, and the risk ratio and its confidence interval. The smaller the range of the confidence interval, the higher the credibility. **(B)** Ranking of the importance of prognostic genes in the machine learning model. The x-axis represents the importance of features, and the y-axis lists the names of various features. **(C)** Risk scores and survival status of TCGA-LUAD patients. The x-axis represents the risk score, which increases from left to right. Upper graph: red dots indicate high - risk patients, and blue dots indicate low - risk patients. Lower graph: red dots indicate deceased patients, and blue dots indicate surviving patients. **(D)** Kaplan-Meier (K-M) curve of the risk model. The x-axis represents overall survival time (days), and the y-axis represents survival probability. Red represents the high - risk group, and blue represents the low - risk group. **(E)** Receiver Operating Characteristic (ROC) curve of the risk model. The x-axis represents specificity, and the y-axis represents sensitivity. The area under the curve (AUC) is a measure of prediction performance, with values closer to 1 indicating better performance. **(F)** Heatmap of the expression levels of prognostic genes in high - and low - risk groups (TCGA-LUAD).

In light of the 29 candidate prognostic genes, a univariate Cox analysis was executed, and 7 genes (P < 0.05, HR ≠ 1) were identified. They were *VDAC1*, *TXNRD1*, *GDF15*, *TRIB3*, *LPL*, *KCNQ1*, and *PKP2*. All of these genes passed the PH test (P > 0.05) and were recorded as prognostic genes (Figure 4A; Supplementary Table S9). Using the seven prognostic genes, an RSF model was constructed to derive the risk score for each LUAD patient. The RSF model unveiled that *VDAC1* had the highest importance in the model (Figure 4B). By the optimal cut-off value of the risk score (54.60984), LUAD patients were sorted into the HRG (n = 195) and the LRG (n = 320). As the risk score rose, the mortality rate of LUAD patients also rose (Figure 4C). The survival rate of the HRG was significantly lower than that of the LRG (P < 0.0001) (Figure 4D). Additionally, the AUC values of the ROC curves for 1, 3, and 5 years were 0.813, 0.815, and 0.809, respectively, all surpassing 0.7 (Figure 4E), indicating that the risk model was relatively accurate for the prognosis of LUAD. Furthermore, the contents of *GDF15*, *LPL*, and *KCNQ1* were higher in the LRG, while the contents of *TXNRD1*, *TRIB3*, *PKP2*, and *VDAC1* were higher in the HRG (Figure 4F). Notably, while several of the identified prognostic genes—including VDAC1, GDF15, and TXNRD1—have been previously implicated in various cancers, their coordinated functional roles within the integrated endoplasmic reticulum stress (ERS)–butyrate metabolism (BM) axis in LUAD have not been systematically explored. Through integrative pathway and single-cell analyses, we delineated distinct yet complementary biological assignments for these genes in this specific context: VDAC1, a mitochondrial outer membrane channel, was predominantly associated with mitochondrial adaptation to butyrate-induced metabolic rewiring, suggesting a potential role in modulating shifts in oxidative phosphorylation and ROS production under systemic butyrate signaling; GDF15, a stress-responsive cytokine, exhibited strong correlation with canonical ERS markers (HSPA5, XBP1s) and was primarily induced by proteotoxic stress rather than direct metabolic regulation, suggesting its potential involvement as a downstream component of the UPR; TXNRD1 and TRIB3 bridged redox homeostasis and ER stress, while LPL and KCNQ1 reflected alterations in lipid catabolism and ion flux linked to butyrate responsiveness. Critically, these genes formed a highly co-expressed module (WGCNA, |cor| > 0.7, P < 1 × 10^−10^) whose combinatorial expression pattern significantly outperformed any single gene in predicting overall survival (AUC_5__γear_ = 0.81 vs. <0.65 for individual genes). This underscores that the novelty of our findings lies not in gene discovery *per se*, but in recontextualizing known molecules within a unified ERS–BM regulatory network specific to LUAD. The risk model was substantiated in GSE31210, and similar results were gained (Supplementary Figure S5), indicating that the risk model constructed in this study was reliable.

### 3.5 Prognostic genes were expressed differently in disease and control samples and were associated with survival in LUAD patients

**FIGURE 5:**
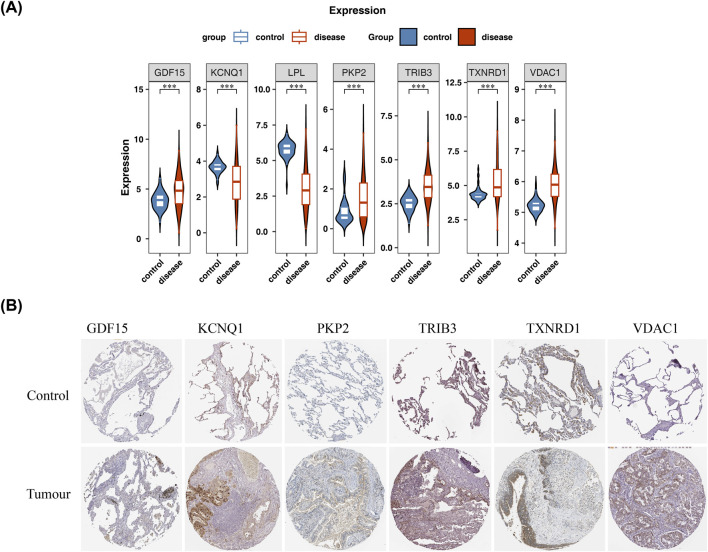
Analysis of gene and protein expression of key genes. **(A)** Gene expression analysis. Blue represents the control group and red represents the disease group. * indicates p < 0.05,** indicates p < 0.01, and *** indicates p < 0.001. **(B)** Protein expression analysis.

Expression analysis unveiled that *VDAC1*, *TXNRD1*, *GDF15*, *TRIB3*, and *PKP2* were highly expressed in disease samples, while *KCNQ1* and *LPL* were highly expressed in control (Figure 5A). To provide tissue-level validation of our findings, we analyzed protein expression data from the Human Protein Atlas (HPA) database. Consistent with our mRNA results, immunohistochemistry (IHC) data from HPA showed higher protein expression of *VDAC1, GDF15*, and PKP2 in LUAD tumor tissues compared to normal lung tissues (Figure 5B). Data for LPL and TRIB3 were not available in the HPA pathology atlas for LUAD. Regrettably, data for *LPL* could not be retrieved. The disease samples in TCGA-LUAD were sorted into HEG and LEG in conformity with the optimal threshold of the expression of each gene, and a K-M survival analysis was applied. The results unveiled that the survival rate of the HEG of *VDAC1*, *PKP2*, *TRIB3* and *TXNRD1* was signally lower than that of the LEG, while the survival rate of the LEG of *KCNQ1*, *GDF15* and *LPL* was lower (P < 0.05) (Supplementary Figure S6).

### 3.6 Independent prognostic factors had good predictive power for the survival of LUAD patients

**FIGURE 6:**
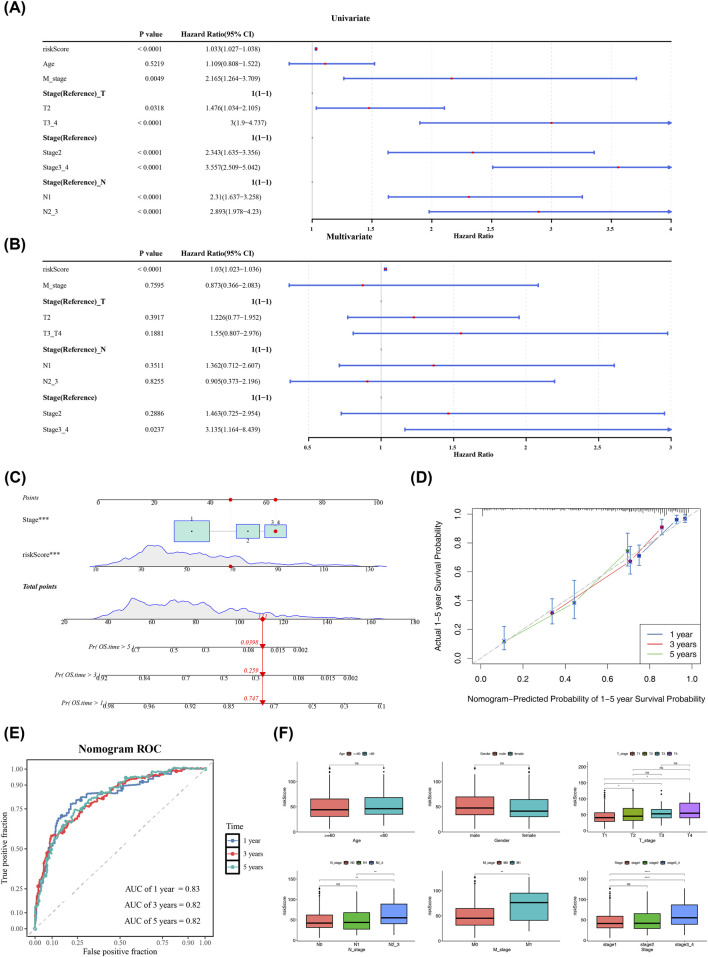
Independent prognostic analysis and nomogram construction. **(A)** Univariate COX forest plot. From left to right are the prognosis - related genes, the corresponding P value, and the risk ratio and its confidence interval. The smaller the range of the confidence interval, the higher the credibility. **(B)** Multivariate Cox regression analysis. **(C)** Nomogram model (TCGA-LUAD). The first part is “points,” indicating the score for a single item when the risk score takes a certain value; the second part is “variables.” The range of the line segment after the variable represents the total contribution of the variable to the outcome event, and the scale on the line segment indicates different values of the variable. The third part is “total points,” representing the total score of individual item scores based on variable values. The fourth part is the predicted survival probability at 1, 3, and 5 years, which shows the survival probability converted from the patient’s total score. **(D)** Calibration curve of the nomogram (TCGA-LUAD). The x-axis represents the predicted probability of the event, and the y-axis represents the actual observed probability of the event, with ranges from 0 to 1. Blue, red, and green represent the predicted survival rates at 1, 3, and 5 years, respectively. The closer the slope is to 1, the better the accuracy of the model’s predictions. **(E)** ROC curve of the nomogram. **(F)** Differences in risk scores across clinical characteristics. The differences between high - and low - risk groups for various clinical characteristics are represented by P values. “****” indicates P < 0.0001, “***” P < 0.001, “**” P < 0.01, “*” P < 0.05, and “ns” indicates P > 0.05.

Univariate Cox regression analysis identified both the risk score and clinical stage as significant prognostic factors for overall survival (OS) in LUAD patients (Figure 6A). To determine whether the risk score provides prognostic information independent of established clinical factors, we constructed a multivariate Cox proportional hazards model that included the risk score, age, gender, and TNM stage. The results demonstrated that the risk score remained a highly significant independent predictor of OS after full adjustment for these clinical covariates [Hazard Ratio (HR) = (1.03), 95% Confidence Interval (CI): (1.023)–(1.036), P < 0.001] (Figure 6B; Supplementary Table S15). Clinical stage was also confirmed as an independent prognostic factor in this model. In light of these two independent prognostic factors, a nomogram model was formulated to predict the 1, 3, and 5-year OS time of LUAD patients (Figure 6C). The slope of the calibration curve of the nomogram was close to 1 (Figure 6D), indicating that the nomogram model constructed in this study had good prediction accuracy. The ROC curves of the nomogram unveiled that the AUC values for 1, 3, and 5 years were 0.83, 0.82, and 0.82, respectively, all of which were surpassing 0.7 (Figure 6E), demonstrating that the nomogram had a high level of prediction accuracy. Moreover, there were striking differences in risk scores among the four clinical subgroups of stage, T_stage, N_stage, and M_stage (P < 0.05) (Figure 6F). In summary, the nomogram model constructed in this study had high prediction accuracy.

### 3.7 GESA in different risk groups and different prognostic genes

**FIGURE 7:**
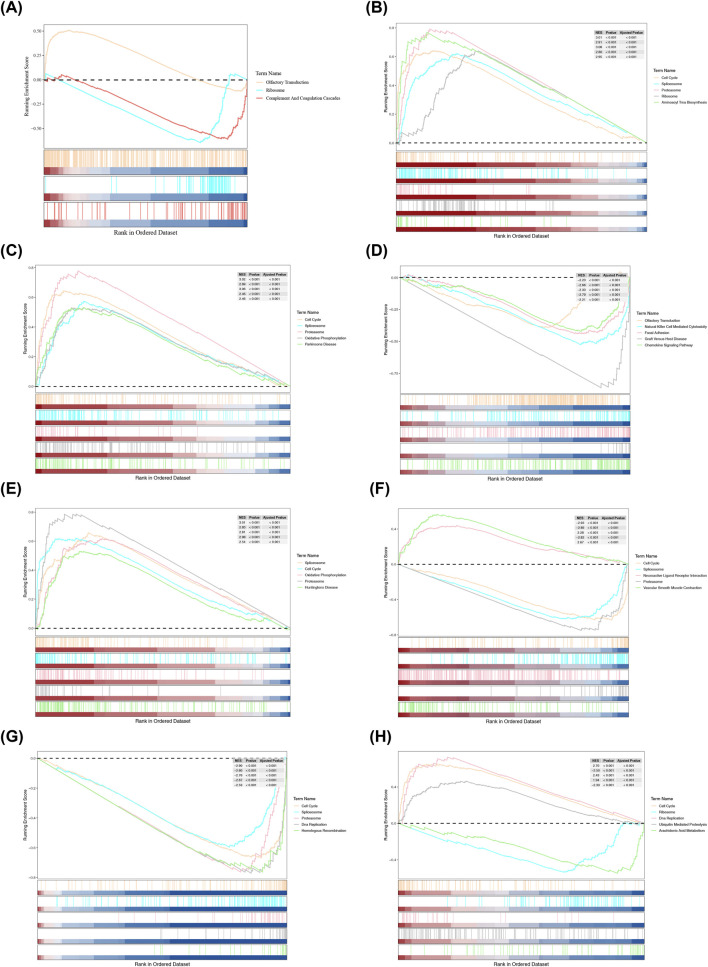
GSEA enrichment analysis. **(A)** GSEA analysis of prognostic genes. The first section shows gene enrichment score (ES) lines. The ES is the peak value in the plot, representing the degree of gene - set enrichment. A positive ES indicates the gene set is enriched in the front of the ranked list and is positively correlated with the gene - set phenotype. A negative ES suggests the gene set is enriched in the rear of the ranked list and is negatively correlated with the phenotype. The x-axis represents genes in the gene set, corresponding to the barcode - like vertical lines in the second section. The second section represents “Hits,” each vertical line corresponding to a gene in the gene set. The third section is the ranked list metric plot, showing the distribution of all gene ranks. **(B)** GSEA analysis of *VDAC1*. **(C)** GSEA analysis of *TXNRD1*. **(D)** GSEA analysis of *GDF15*. **(E)** GSEA analysis of *TRIB3*. **(F)** GSEA analysis of *LPL*. **(G)** GSEA analysis of *KCNQ1*. **(H)** GSEA analysis of *PKP2*.

GSEA analysis identified three significantly enriched pathways in both the HRG and the LRG. Olfactory transduction was significantly enriched in the HRG, while ribosome and complement, and coagulation cascades were significantly enriched in the LRG (Figure 7A). This manifested that there were striking differences in biological functions between the HRG and the LRG. Olfactory transduction might be associated with specific physiological activities of the HRG, while the LRG was likely to be more involved in processes related to ribosome and complement-coagulation. Furthermore, the GSEA analysis executed on the prognostic genes evidenced that the seven prognostic genes were significantly co-enriched in pathways such as cell cycle, ribosome, and DNA replication (Figures 7B–H; Supplementary Table S11). This meant that these seven prognostic genes might jointly participate in crucial biological processes such as cell-cycle regulation, ribosome function, and DNA replication through Potential functional link, exerting a noteworthy impact on the prognosis of LUAD.

### 3.8 Different immune microenvironments and gene mutations within HRG and LRG

**FIGURE 8:**
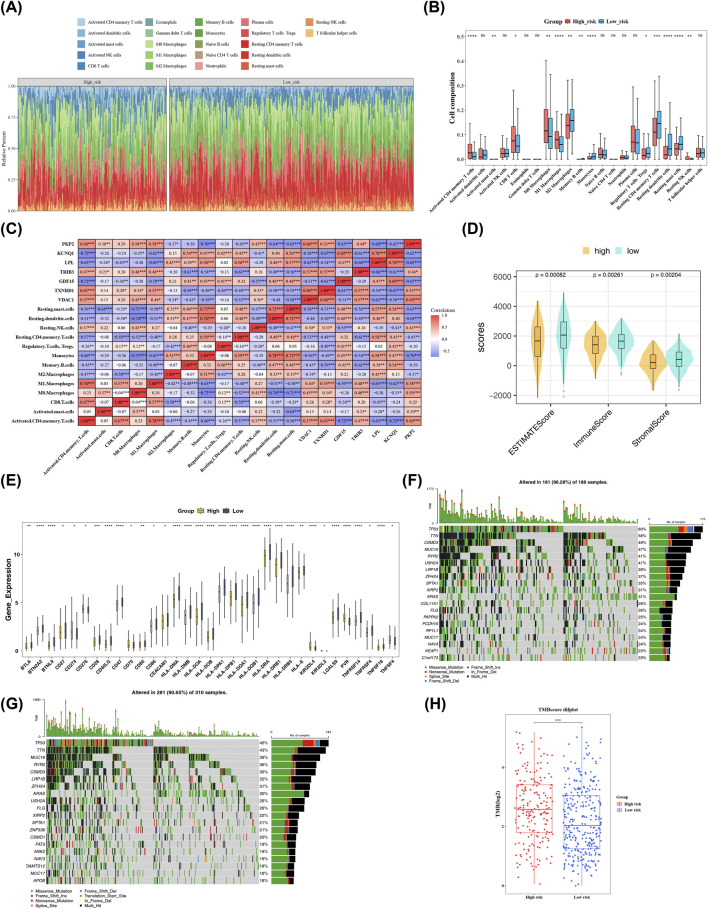
Immune infiltration analysis. **(A)** Relative abundance of immune cells. Different colors represent different immune cells, and the proportion of each color represents the proportion of the corresponding immune cell in the sample, with each line representing a sample. **(B)** Differences in immune cell infiltration between high - and low - risk groups. The x-axis shows different immune cells, and the y-axis shows immune cell infiltration scores. A higher score indicates more immune cell infiltration. **(C)** Correlation analysis heatmap. Red indicates positive correlation, with stronger correlations shown in deeper red. Blue indicates negative correlation, with stronger correlations in deeper blue. Numbers show correlation coefficients. **(D)** Comparison of stromal score, immune score, and ESTIMATE score between high - and low - risk groups. The x-axis represents ESTIMATE, immune, and stromal scores, and the y-axis represents the scores. Yellow shows the high - risk group, and green shows the low - risk group. **(E)** Expression of immune checkpoints in the low - risk group. Blue represents the high - risk group, and yellow represents the low - risk group. **(F)** Top 20 genes with the highest mutation frequency in the high - risk group. The upper graph shows the high - risk group, and the lower graph shows the low - risk group. The y-axis lists genes, and different colors on the x-axis represent different mutation types. **(G)** Top 20 genes with the highest mutation frequency in the low - risk group. **(H)** Differences in TMB between high - and low - risk groups. The x-axis represents risk groups, and the y-axis represents the logarithm (log_10_) of TMB. * means P < 0.05, **P < 0.01, ***P < 0.001, and ****P < 0.0001.

The immune infiltration within the HRG and the LRG was shown in Figure 8A. On one hand, there were 13 types of differential immune cells, such as activated CD4 memory T cells, resting NK cells, within the HRG and the LRG (P < 0.05) (Figure 8B). There was a strong positive connection within activated CD4 memory T cells and M1 macrophages (cor = 0.78, P < 0.001), and a strong negative connection within resting dendritic cells and M0 macrophages (cor = −0.74, P < 0.001). Additionally, *TXNRD1* had a strong positive connection with activated CD4 memory T cells (cor = 0.63, P < 0.01), while *KCNQ1* and *GDF15* had a strong negative connection with activated CD4 memory T cells (cor = −0.71, P < 0.001) (Figure 8C). Interestingly, the immune score (P = 0.00261), stromal score (P = 0.00204), and ESTIMATE score (P = 0.00082) of the LRG were signally higher than those of the HRG (Figure 8D). Moreover, there were 33 differentially expressed immune checkpoints, such as BTLA, BTN2A2, and BTNL9, within the HRG and the LRG (P < 0.05) (Figure 8E). These results not only revealed the underlying mechanisms of the differences in immune states within the HRG and the LRG in LUAD but also opened up new ideas for immunomodulatory therapies and prognostic evaluation of LUAD, showing great potential for clinical translation.

On the other hand, gene mutation analysis revealed that TP53 and TTN had a high mutation frequency in both the HRG and the LRG. Moreover, the most common mutation type in both the HRG and the LRG was a missense variant (Figures 8F,G). Additionally, the TMB score in the HRG was significantly higher than that in the LRG (P < 0.001) (Figure 8H). These characteristics of gene mutations and the differences in TMB scores provided crucial clues for understanding the tumorigenesis and development mechanisms of the HRG and the LRG, and were expected to contribute to the exploration and development of targeted therapeutic strategies.

### 3.9 Molecular regulatory networks were constructed, and potential drugs were predicted

**FIGURE 9:**
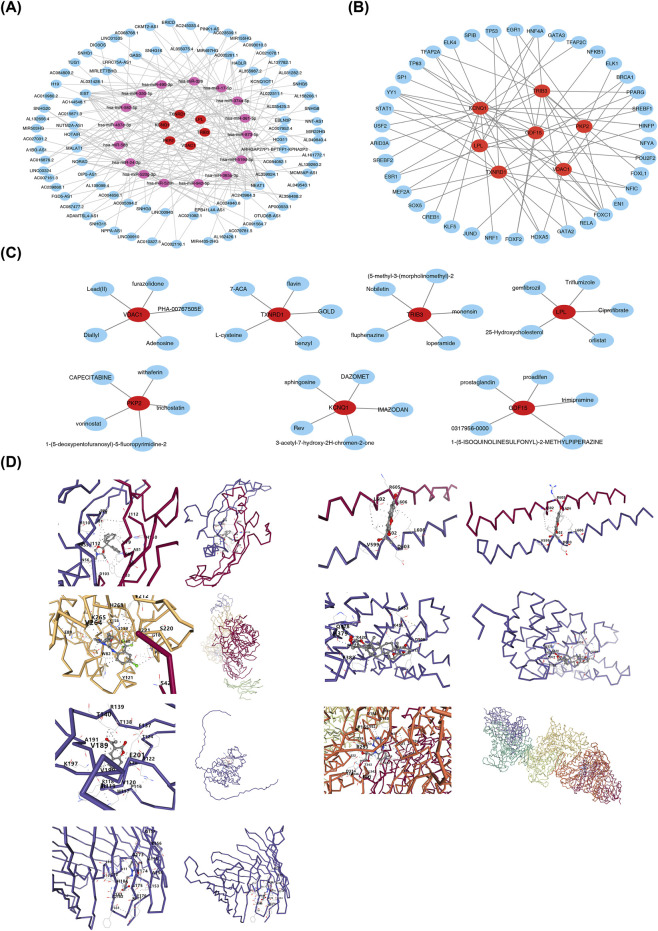
Construction of molecular regulatory networks. **(A)** lncRNA-miRNA-mRNA network. Red circles represent prognostic genes, purple circles are predicted miRNAs, and blue circles are lncRNAs. **(B)** TF-mRNA network. **(C)** Target - drug prediction network. Red circles indicate key genes, and blue circles represent drugs. **(D)** Prognostic genes and molecular docking. The left part shows the overall effect of protein - 3D - structure docking, and the right part displays the magnified view of the docking site. Different colors represent different protein chains.

The lncRNA–miRNA–mRNA network revealed 192 regulatory relationships in LUAD, including HAGLR–hsa-miR-17-5p–VDAC1 and XIST–hsa-miR-487a-3p–PKP2 (Figure 9A). Concurrently, transcription factors (TFs) capable of targeting the prognostic genes—such as FOXC1, HNF4A, and HOXA5—were predicted, and a TF–mRNA regulatory network comprising 45 nodes and 68 edges was constructed (Figure 9B).

Furthermore, potential candidate compounds were computationally predicted for each of the seven prognostic genes using the DSigDB database. The top five compounds ranked by prediction score for each gene are displayed in Figure 9C. Molecular docking analysis indicated that each prognostic gene exhibited high binding affinity with its top-scoring compound (Figure 9D; Table 1). It should be emphasized that these drug–target interactions are based solely on *in silico* predictions, and their biological relevance in LUAD cells remains to be experimentally validated.

**TABLE 1:** Molecular docking result.

Prognostic gene (3D structure name)	Drug	Binding energy (KJ/mol)
*GDF15* (5VT2)	Proadifen	−5.0
*PKP2* (3TT9)	Withaferin	−8.2
*VDAC1* (2JK4)	Furazolidone	−8.2
*TXNRD1* (2CFY)	Flavin	−9.1
*LPL* (6E7K)	Triflumizole	−7.6
*KCNQ1* (3BJ4)	3-acetyl-7-hydroxy-2H-chromen-2-one	−4.6
*TRIB3* AF-Q96RU7-F1-v4)	(5-methyl-3-(morpholinomethyl)-2,3-dihydro-[1,4]oxazino[2,3,4-hi]indol-6-yl)(naphthalen-1-yl)methanone	−6.6

### 3.10 Eight cell types were summarized and macrophages were ascertained as the key cells

**FIGURE 10:**
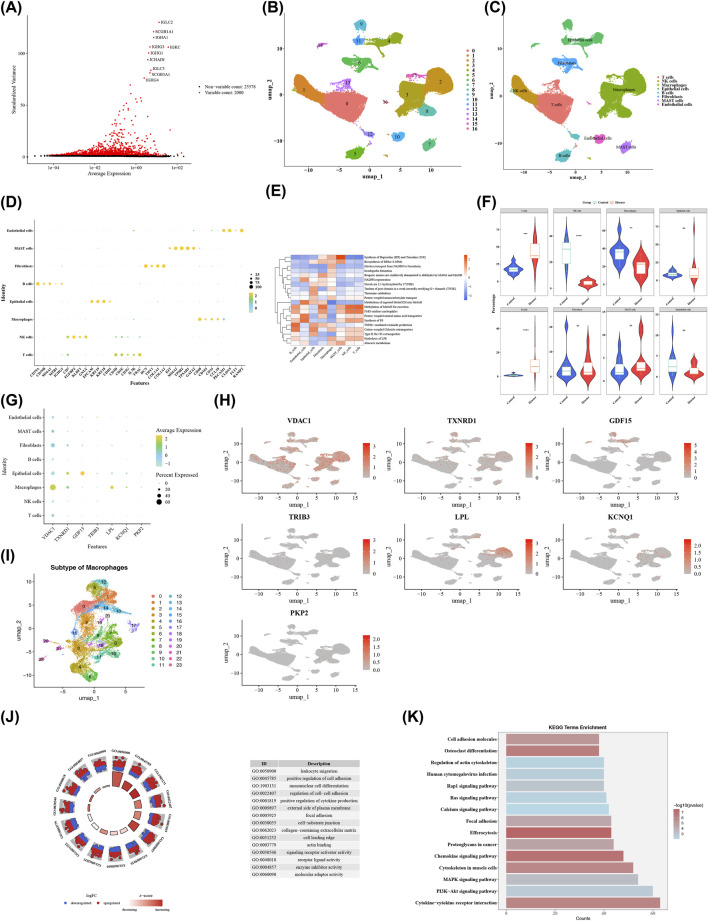
Single - cell data analysis. **(A)** Selection of highly variable genes. The x-axis represents gene expression levels, and the y-axis represents the high variability of genes. Red dots indicate the top 2,000 highly variable genes. **(B)** Cell UMAP clustering. Each color represents a cell type, as indicated in the legend. **(C)** Cell UMAP annotation. **(D)** Expression of marker genes in cell clusters. **(E)** Functional enrichment of cell clusters. The x-axis represents cells, and the y-axis represents pathways. Colors indicate enrichment levels, with blue for negative correlation and red for positive correlation. **(F)** Differences in cell proportions between disease and control samples. Blue represents the control group, and red represents the disease group. * means P < 0.05, **P < 0.01, ***P < 0.001, and ****P < 0.0001. **(G)** Expression of biomarkers. **(H)** Expression of prognostic genes in the cell UMAP clustering plot. Darker colors indicate higher expression levels. **(I)** Dimensionality reduction and clustering analysis; annotation of key cell subclusters. **(J)** GO enrichment analysis of selected genes. **(K)** KEGG enrichment analysis of candidate genes. The x-axis is labeled “Counts,” representing the number of genes. The y-axis lists various KEGG terms.

After single-cell transcriptome analysis, a total of 81,440 cells and 27,578 genes met the criteria. Initially, we generated visualizations of nFeature_RNA, nCount_RNA, and percent_mt before and after quality control (Supplementary Figures S7A,B). For subsequent analysis, 2,000 HVGs were selected, and the top 10 genes with the most noteworthy changes in content were highlighted (Figure 10A). After applying PCA, the top 20 principal components were selected for further analysis (see Supplementary Figures S7C,D). Subsequently, the clustering analysis ascertained 17 different cell groups (Figure 10B). To ensure robust and interpretable cell type annotation, we integrated computational prediction with manual curation based on canonical lineage-defining markers. The final annotation identified eight major cell types: malignant epithelial cells, T cells, B cells, NK cells, macrophages, dendritic cells, fibroblasts, and endothelial cells (Figures 10C,D).

This annotation was rigorously validated through comprehensive visualization. A DotPlot (Figure 10E) clearly demonstrates the distinct expression patterns of key marker genes across all clusters: epithelial identity is marked by high EPCAM and KRT18; myeloid lineages (macrophages, dendritic cells) by CD68, CD163, and LYZ; T/NK cells by CD3D and NCR1; fibroblasts by COL1A1 and DCN; and endothelial cells by PECAM1 and VWF. Consistently, FeaturePlots (Supplementary Figure S9) show the spatially restricted expression of these markers within their respective UMAP domains, confirming the fidelity of our annotation.

The list of marker genes was shown in Supplementary Table S12. These eight cell types were signally enriched in biological pathways such as integration of hepoxilins (HX) and trioxilins (TrX) and electron transport from NADPH to ferredoxin (Figure 10E). To address the cell-type-specific nature of ERS and metabolic programs, we quantified their activity in each annotated lineage. Using the AddModuleScore algorithm, we calculated ERS and butyrate metabolism (BM) scores for every single cell. Strikingly, the distribution of these scores was highly heterogeneous across the tumor microenvironment (Figures 10F,G).

ERS activity was predominantly enriched in myeloid cells, with macrophages exhibiting the highest median ERS score among all cell types, followed by dendritic cells. In contrast, malignant epithelial cells, T cells, and fibroblasts showed relatively lower ERS activity.

Conversely, butyrate metabolism (BM) activity was most pronounced in malignant epithelial cells, which displayed the highest median BM score. Myeloid cells (macrophages and dendritic cells) also exhibited moderate BM activity, while other immune and stromal populations showed minimal scores.

This clear segregation of ERS and BM activities—ERS being a hallmark of tumor-associated myeloid cells and BM being a feature of malignant epithelium—highlights the functional specialization of different cellular compartments within the LUAD microenvironment and provides a critical context for interpreting our subsequent analyses.

Moreover, the proportions of these eight cell types differed between the disease samples and the control samples. T cells had the highest proportion in the disease samples, while macrophages had the highest proportion in the control samples (see Supplementary Figure S7E). There were striking variations in the proportions of T cells, NK cells, Macrophages, and B cells within the disease samples and the control samples (P < 0.05) (Figure 10F). Meanwhile, *VDAC1*, *TXNRD1*, and *LPL* were all highly expressed in macrophages. Therefore, macrophages were regarded as the key cells in this study (Figures 10G,H). To gain deeper insights into the functional heterogeneity of these key cells, we performed a focused analysis on the classical M1 (pro-inflammatory) and M2 (anti-inflammatory) macrophage subtypes. A detailed examination of the seven prognostic genes revealed distinct and subtype-specific expression patterns (Supplementary Figure S11). In M1 macrophages, VDAC1 exhibited the highest cellular prevalence (largest dot), albeit with relatively low average expression intensity (cooler color). Conversely, PKP2 showed high average expression (warmer color) but was present in only a very small fraction of M1 cells (smallest dot). The remaining genes displayed low levels of both expression and cellular prevalence. In stark contrast, within M2 macrophages, both VDAC1 and LPL were characterized by high average expression (warm colors) and a relatively high proportion of positive cells (larger dots). PKP2, however, was barely detectable in M2 macrophages, showing both low expression and minimal cellular prevalence. This clear dichotomy in the expression profiles of VDAC1, LPL, and PKP2 between M1 and M2 subtypes underscores a profound functional divergence and suggests their potential roles in shaping the polarization state of tumor-associated macrophages (TAMs) in LUAD.Macrophages were further annotated into 24 sub-classes (Figure 10I). Additionally, there were 2223 DEGs in macrophages. These DEGs were significantly enriched in 2193 GO terms and 335 KEGG pathways (Figures 10J,K; Supplementary Tables S13, S14).

### 3.11 Functional reprogramming of macrophages under high endoplasmic reticulum stress

To address the need for deeper, cell-type-resolved pathway analysis, we dissected the transcriptional programs associated with varying levels of ERS specifically within the macrophage compartment. By calculating an ERS activity score for each individual macrophage, we stratified the population into ERS-high (n = 11,210) and ERS-low (n = 11,211) subgroups using the median score as the cutoff.

Comparative analysis between these two states identified 285 significantly differentially expressed genes (DEGs; |log_2_FC| > 1, adjusted p < 0.05), of which 148 were upregulated and 137 were downregulated in the ERS-high group. Subsequent Gene Set Enrichment Analysis (GSEA) revealed a striking pattern: all seven significantly enriched pathways (BH-adjusted p < 0.05) were negatively enriched (Normalized Enrichment Score, NES <0) in the ERS-high macrophages (Supplementary Figure S8).

These downregulated pathways were predominantly related to immune and inflammatory functions, including “positive regulation of cytokine production” (GO:0001819), “immune response” (GO:0006955), “TNFα signaling via NF-κB” (M5892), “cytokine production” (GO:0001816), “regulation of immune response” (GO:0050776), “positive regulation of gene expression” (GO:0010628), and “cell activation” (GO:0001775). This coordinated downregulation strongly suggests that a high ERS state drives macrophages towards an immunosuppressive or functionally impaired phenotype, characterized by attenuated pro-inflammatory responses.

Notably, pathways of interest in our broader study context, such as “bone remodeling,” “extracellular matrix organization,” and “butyrate metabolism,” did not show statistically significant enrichment in this macrophage-specific comparison. This finding indicates that the primary transcriptional consequence of elevated ERS in LUAD-associated macrophages is the modulation of immune-inflammatory circuits, rather than these other metabolic or structural processes.

### 3.12 Tissue-level protein validation using the Human Protein Atlas (HPA)

To complement our cell line-based mRNA validation and address the clinical relevance of our prognostic signature, we performed an in-depth analysis of protein expression at the tissue level using data from the Human Protein Atlas (HPA) database. We focused on the five genes (VDAC1, TXNRD1, GDF15, KCNQ1, PKP2) for which relative protein abundance data (normalized Relative Protein eXpression, nRPX) from mass spectrometry (CPTAC) were available for both LUAD tumor and normal lung tissue samples.

Using the Wilcoxon rank-sum test, we compared the nRPX values between tumor and normal groups. The analysis revealed that VDAC1, GDF15, and PKP2 exhibited significantly higher protein expression levels in LUAD tumor tissues compared to normal lung tissues (P < 0.05, P < 0.01, and P < 0.001, respectively) (Supplementary Figure S10). In contrast, TXNRD1 and KCNQ1 showed no statistically significant difference in protein abundance between the two groups. These results provide crucial orthogonal validation at the protein level within a clinical tissue context, strengthening the association of our identified genes with LUAD pathogenesis.

### 3.13 Cell communication and pseudo-time analysis of macrophages

**FIGURE 11:**
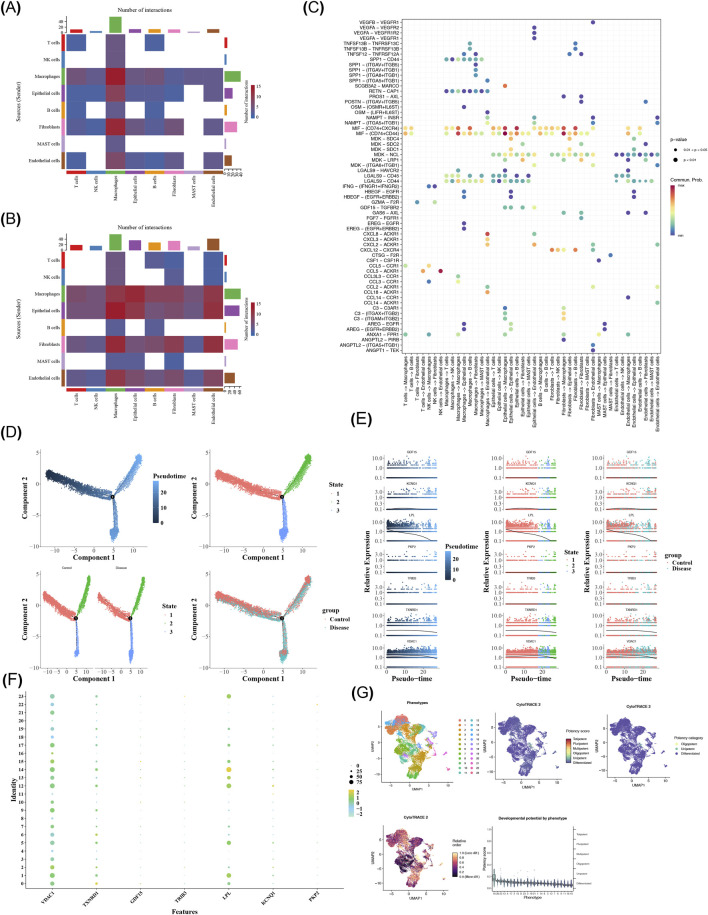
Cell communication and trajectory analysis. **(A)** Cell - communication heatmap between all cell types in the control group. **(B)** Cell - communication heatmap between all cell types in the disease group. **(C)** Receptor - ligand communication between different cell types. They-axis shows different receptor - ligand pairs, and the x-axis represents different cell - cell relationships. Colors range from blue to red, with redder colors indicating stronger relationships. **(D)** Cell trajectory analysis. The left graph shows cell trajectories, and the right graph divides cell - development states into three phases, each represented by a different color. **(E)** Dynamic gene - expression profiles. The y-axis represents relative gene - expression levels, and the x-axis shows the expression of prognostic genes at different time stages. **(F)** Expression of prognostic genes in key cell subclusters. **(G)** Differentiation of key cells. Different colors represent different cell subtypes.

Cell communication analysis unveiled that macrophages interacted more frequently with epithelial cells in the disease group contrasted to the control group (Figures 11A,B). In addition, the interactions within epithelial cells and macrophages, as well as within fibroblasts and macrophages, could occur through MIF-(CD74^+^CD44) (Figure 11C). Macrophages were classified into three different branches and three different cell populations In accordance with the cell development state. The disease group had more cells than the control group at the initial stage of differentiation (Figure 11D). Besides, the *VDAC1*, *TXNRD1*, and *LPL* genes were highly expressed at the initial stage of cell differentiation, while the expression of *GDF15*, *TRIB3*, *KCNQ1*, and *PKP2* unveiled no obvious changes during the cell differentiation process (Figure 11E). Notably, *LPL* was highly expressed in 14 subpopulations of macrophages (Figure 11F). CytoTRACE analysis revealed that the cell subclass 16 of macrophages was the initial stage of differentiation (Figure 11G).

### 3.14 Significantly differential expression of prognostic genes

**FIGURE 12:**
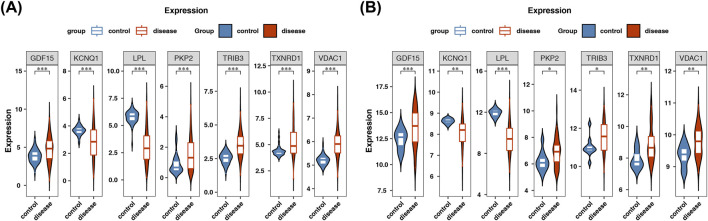
Differential expression of prognostic genes in LUAD and normal in TCGA-LUAD and GSE116959 datasets. **(A)** Differences in the expression of prognostic genes in the TCGA-LUAD dataset. Red represents the control group, and blue represents the LUAD group. Statistical significance annotations: *p < 0.05, **p < 0.01, ***p < 0.001. **(B)** Differences in the expression of prognostic genes in the GSE116959 dataset. Red represents the control group, and blue represents the LUAD group. Statistical significance annotation: ***p < 0.001.

The expression analysis showed that in both datasets TCGA-LUAD and GSE116959, 7 prognostic genes were significantly differentially expressed. Compared with the normal group, *GDF15*, *PKP2*, *TRIB3*, *TXNRD1* and *VDAC1* were significantly upregulated in the tumor group, while *KCNQ1* and *LPL* were significantly downregulated (Figure 12). The consistent expression of prognostic genes across multiple datasets also indicated the stability of their expression.

### 3.15 Validation of prognostic gene expression

**FIGURE 13:**
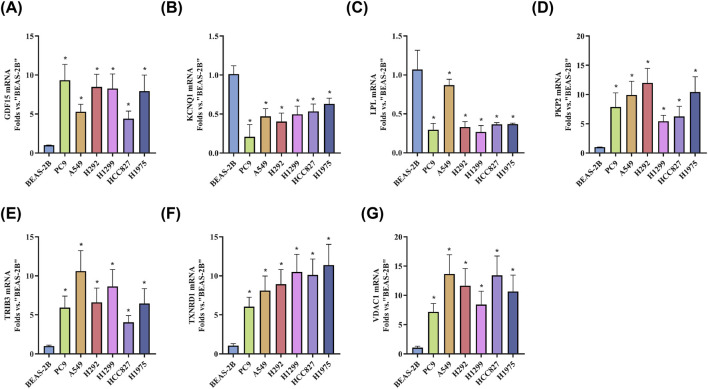
RT-qPCR validation of the seven prognostic genes in lung cancer cell lines. The mRNA expression levels of the seven prognostic genes were measured in a panel of NSCLC cell lines (PC9, A549, H292, H1299, HCC827, H1975) and compared to the normal human bronchial epithelial cell line BEAS-2B. Data are presented as mean ± SEM from three independent biological replicates. Statistical significance was determined using an unpaired, two-tailed Student’s t-test. *P < 0.05, **P < 0.01, ***P < 0.001.

RT-qPCR validation results demonstrated that all seven prognostic genes exhibited differential expression in lung cancer cell lines (PC9, A549, H1299, H292, HCC827, H1975) when compared with the normal human lung epithelial cell line BEAS-2B. Specifically, *GDF15*, *PKP2*, *TRIB3*, *TXNRD1* and *VDAC1* were significantly upregulated in lung cancer cells: the expression level of *GDF15* was 11-fold higher in H1299 cells, while that of *PKP2* and *VDAC1* reached 15-fold and 17-fold higher respectively in H292 cells (Figures 13A,D–G). Meanwhile, the expression patterns of *KCNQ1* and *LPL* were consistent with those observed in public datasets, with statistically significant differences detected (Figures 13B,C).

## 4 Discussion

LUAD, the most prevalent subtype of NSCLC (Song et al., 2023). Despite substantial progress in targeted therapy and immunotherapy, drug resistance and poor prognosis continue to pose major clinical challenges, highlighting the urgent need for novel biomarkers and mechanistic insights (Al-Dherasi et al., 2021). Emerging evidence suggests that ERS and BM play pivotal roles in tumor progression, immune evasion, and therapy resistance; however, their potential functional link in LUAD remain poorly understood (Chen et al., 2024). The rationale for jointly investigating endoplasmic reticulum stress (ERS) and butyrate metabolism (BM) stems from growing evidence of their functional crosstalk in shaping the tumor microenvironment. Butyrate, a microbial metabolite and histone deacetylase (HDAC) inhibitor, exhibits context-dependent modulation of ERS: it can either exacerbate ERS by upregulating UPR markers (e.g., GRP78, CHOP, IRE1α) and inducing autophagy in cancer cells (Zhang J. et al., 2016), or attenuate ERS in inflammatory settings by suppressing pathways such as STING–ER stress. Mechanistically, butyrate-mediated HDAC inhibition alters the acetylation and function of ER chaperones like GRP78, thereby rewiring UPR signaling and cell fate decisions (Chen et al., 2017). Given that both ERS and BM influence immune cell polarization (e.g., macrophages), stromal remodeling, and metabolic adaptation in tumors, their intersection likely constitutes a critical “stress-metabolic” regulatory node in LUAD. Analyzing these processes in isolation would overlook key hub genes operating at this interface—genes that may orchestrate proteostasis, immunometabolism, and therapeutic resistance. Our integrative approach was therefore designed to capture this synergy, enabling the discovery of novel biomarkers and potential targets for immuno-metabolic combination strategies. In this study, we screened out 7 core prognostic genes associated with ERS-BM through integrating multi-dimensional omics data and combining multiple screening strategies such as differential expression analysis, WGCNA, and MR analysis. These genes include *VDAC1*, *TXNRD1*, GDF-15, *TRIB3*, *LPL*, *KCNQ1*, and *PKP2*. A potential concern is that key genes identified in this study—such as VDAC1, GDF15, and TXNRD1—are well-documented in prior cancer literature. However, the conceptual advance of our work resides in the biological context and integrative logic, not in the novelty of individual components. Previous studies have largely treated these genes as isolated entities: VDAC1 in mitochondrial apoptosis (Zhang G. et al., 2016; Liu W. S. et al., 2024), GDF15 in cachexia or TGF-β signaling (Lu et al., 2017), and TXNRD1 in antioxidant defense (Jiang et al., 2024; Huang et al., 2023). These observations, while valuable, remain fragmented and disconnected from upstream metabolic drivers or downstream immune consequences.

In contrast, by anchoring our analysis in the gut–lung axis–mediated butyrate signaling and ERS convergence, we reveal that these genes function as interconnected nodes in a stress-metabolic circuit that collectively shapes LUAD progression. For instance, VDAC1 transitions from a generic mitochondrial pore to a sensor of butyrate-induced metabolic flux, while GDF15 becomes a specific readout of ERS intensity in tumor epithelial cells. Moreover, our multi-omics framework—integrating Mendelian randomization (causal inference), single-cell resolution (cell-type specificity), and risk modeling (combinatorial prognostic power)—demonstrates how these genes synergistically respond to systemic metabolic pressure to modulate the tumor microenvironment and clinical outcomes.

Thus, our study moves beyond single-gene biomarker discovery toward a systems-level understanding of how established cancer-associated molecules acquire new functional identities when embedded in the ERS–butyrate metabolic network—a paradigm shift with implications for precision therapy and combination strategies. These genes exhibit complex molecular regulatory networks during the progression of LUAD.

As a core component of the mitochondrial voltage-dependent anion channel, VDAC1 is highly expressed in LUAD. Its elevated expression is strongly associated with poor prognosis, and it may participate in tumor progression through the regulation of cellular energy metabolism, as suggested by prior studies (Zhang G. et al., 2016; Liu W. S. et al., 2024).

TXNRD1 plays a pivotal role in oxidative stress regulation, with its oncogenic mechanisms exhibiting multidimensional characteristics. In LUAD, high TXNRD1 expression is significantly correlated with adverse clinical outcomes. Previous reports indicate that TXNRD1 may protect tumor cells from radiotherapy- or chemotherapy-induced apoptosis by mitigating ROS-mediated DNA damage (Jiang et al., 2024). Additionally, TXNRD1 has been implicated in promoting inflammatory responses via modulation of the NF-κB signaling pathway, which could contribute to immune suppression in the tumor microenvironment (Huang et al., 2023).

GDF15 is involved in regulating cell differentiation, inflammatory responses, and energy metabolism. In LUAD, elevated GDF15 expression correlates with enhanced invasive and migratory potential of cancer cells, potentially through activation of the TGF-β/Smad signaling pathway, as described in earlier work (Lu et al., 2017).

The TRIB3 gene encodes a pseudokinase that participates in multiple cellular processes and has been linked to various cancers. In LUAD, high TRIB3 expression is associated with unfavorable prognosis, and it may support tumor cell survival and proliferation by promoting ERK and JNK phosphorylation and upregulating HIF-1α-mediated glycolytic reprogramming, based on findings from previous functional studies (Cao et al., 2020; Xing et al., 2020).

LPL, a key enzyme in lipid metabolism, catalyzes the hydrolysis of triglycerides in chylomicrons and very-low-density lipoproteins. Its low expression in LUAD is associated with poor prognosis, possibly reflecting a loss of its putative tumor-suppressive role in maintaining tumor microenvironment homeostasis, which may otherwise help restrain tumor growth and metastasis (Wei et al., 2025).

KCNQ1 is an important ion channel for maintaining cell membrane potential. Both genetic variations and high expression of KCNQ1 have been associated with better prognosis in the TCGA-LUAD cohort. KCNQ1 may also be involved in modulating immune responses within the LUAD tumor microenvironment, thereby influencing disease progression, although the underlying mechanisms remain incompletely understood (Chang et al., 2022).

PKP2, a core component of intercellular desmosomes, contributes to the maintenance of epithelial cell adhesion. In LUAD, higher PKP2 expression is correlated with favorable clinical features and may influence tumor development through modulation of epithelial-mesenchymal transition (EMT) and focal adhesion dynamics, as previously reported (Luo et al., 2023).

Collectively, these seven genes show consistent associations with key biological processes—including metabolic regulation, stress responses, invasion/metastasis, and immune modulation—in LUAD. While their individual roles have been suggested in prior studies, our integrative analysis highlights their co-occurrence within ERS- and butyrate metabolism-related networks, warranting further functional investigation. The robustness of our findings is supported by a convergent four-layered evidence framework. First, single-cell transcriptomics pinpointed macrophages as a key cellular hub where several of these prognostic genes (notably VDAC1, TXNRD1, and LPL) are highly co-expressed, particularly during early differentiation stages, suggesting a cell-type-specific regulatory context. Second, the differential expression of all seven genes was consistently validated across multiple independent cohorts (TCGA-LUAD and GSE116959) at the mRNA level and further confirmed by RT-qPCR assays in a panel of NSCLC cell lines compared to normal bronchial epithelial cells, demonstrating their stability across different data sources and experimental platforms. Third, survival analysis robustly linked the expression levels of each gene, and more powerfully their combined risk score, to patient overall survival, establishing their clinical relevance as prognostic indicators. Fourth, and most critically for inferring potential causality, Mendelian randomization (MR) analysis provided genetic evidence supporting a causal relationship between the expression of these genes and LUAD risk, thereby strengthening the hypothesis that they are not merely correlative biomarkers but potentially active players in disease pathogenesis. This multi-faceted convergence of evidence underscores the significance of the identified ERS-BM gene signature in LUAD.

While our integrative analysis identifies a set of shared prognostic genes linking endoplasmic reticulum stress (ERS) and butyrate metabolism (BM), it is crucial to acknowledge that the direct mechanistic crosstalk between these two pathways was not experimentally validated in this study. Nonetheless, the observed potential functional link is strongly supported by a convergent quartet of bioinformatic evidence: (i) single-cell co-expression, where key genes such as VDAC1, TXNRD1, and LPL are concurrently and highly expressed within the same macrophage subsets in the LUAD TME (Figures 10G,H); (ii) bulk-tissue co-variation, demonstrated by the consistent differential expression of all seven prognostic genes across multiple independent cohorts (TCGA-LUAD, GSE116959) and NSCLC cell lines (Figures 12, 13); and (iii) genetic causality inferred from Mendelian randomization, which prioritized these genes as potential drivers of LUAD risk rather than mere correlative markers.

This proposed link is further substantiated by emerging literature from related fields. Recent studies have shown that butyrate, beyond its role as an HDAC inhibitor, can directly modulate ER homeostasis. For example, in colonic epithelial cells, butyrate was found to enhance the acetylation of the ER chaperone GRP78, thereby fine-tuning the unfolded protein response (UPR) and promoting cell survival under stress (Aliyu et al., 2023). Conversely, activation of the ERS sensor IRE1α has been reported to rewire cellular metabolism, including mitochondrial fatty acid oxidation—a process intimately linked to butyrate utilization, (Liu S. J. et al., 2024). Although these findings originate from non-pulmonary contexts, they provide a compelling biological rationale for the ERS-BM interplay we infer in LUAD.

Further GSEA showed significant differences in the signaling pathways enriched between the HRG and LRG in LUAD. The HRG was specifically enriched in the olfactory transduction pathway. Activation of olfactory receptors (ORs) such as OR2J3 leads to an increase in intracellular Ca^2+^ concentration, which in turn triggers phosphorylation of components of the ERK signaling pathway to promote tumor cell proliferation. Additionally, research has demonstrated that lower OR51E2 expression is significantly associated with better survival rates, suggesting that the expression levels of ORs in lung cancer patients have potential prognostic evaluation value (Chung et al., 2022). The LRG was enriched in the ribosome pathway and the complement-coagulation cascade pathway. Activation of the ribosome pathway in the LRG indicates restricted protein synthesis, which may delay tumor progression by inhibiting MYC-driven ribosome biogenesis. Studies have found that *KCNQ1* downregulates ribosomal protein S6 kinase (S6K) by inhibiting the mTORC1 signaling pathway, thereby limiting the translational capacity of tumor cells (Jiao et al., 2024). In addition, *TXNRD1* protects ribosomes from oxidative damage and maintains their homeostasis by scavenging ROS (Rong et al., 2021). The complement-coagulation cascade pathway is closely linked to the statute of the tumor immune microenvironment. Studies have found that the complement system exhibits bidirectional regulatory properties during tumor immune responses: on one hand, complement activation can trigger effective antitumor cytotoxic responses and exert immune surveillance functions; on the other hand, complement proteins such as C3 and C3a can inhibit antitumor T cell responses by recruiting myeloid-derived suppressor cells, M2 macrophages, or Treg cells (Gustavsson et al., 2022). Our identification of potential candidate compounds—such as furazolidone for VDAC1 and flavin derivatives for TXNRD1—is based exclusively on *in silico* molecular docking and database mining. It is important to note that computational docking is a standard initial screening tool in early drug discovery, primarily used to prioritize lead compounds for experimental follow-up. The high binding affinities predicted here should therefore be interpreted as hypothesis-generating, not as evidence of therapeutic efficacy. The actual biological activity, cytotoxicity, and on-target effects of these compounds in LUAD models remain unverified and require rigorous *in vitro* validation.

Single-cell clustering analysis revealed significant heterogeneity of macrophages in LUAD tissues, which could be further divided into pro-inflammatory (M1-like) and anti-inflammatory (M2-like) subpopulations. Notably, *VDAC1*, *TXNRD1*, and *LPL* were highly expressed in macrophages at early differentiation stages, suggesting that these genes may regulate the polarization direction of macrophages. Previous studies have shown that *VDAC1* promotes M2 polarization through mitochondrial oxidative phosphorylation (OXPHOS) (Song et al., 2022), while *LPL* reverses this process by inhibiting fatty acid synthase (FASN) (Chang et al., 2019). Additionally, high expression of *TRIB3* may enhance the survival ability of macrophages in hypoxic environments by activating the HIF-1α pathway (Liu C. et al., 2021), thereby promoting the formation of an immunosuppressive microenvironment.

We found that macrophages and epithelial cells communicated via the MIF-(CD74^+^CD44) ligand-receptor pair, and this signaling axis was significantly enhanced in the disease group. The MIF-CD74 pathway can activate PI3K/AKT and NF-κB signaling (Luo et al., 2023), thereby promoting epithelial-mesenchymal transition (EMT) and angiogenesis. Moreover, macrophages with high *GDF15* expression may weaken anti-tumor immune responses by inhibiting NK cell activity (Liu L. et al., 2024). These results are consistent with recent studies showing that tumor-associated macrophages (TAMs) reshape the stroma and assist tumor immune escape by secreting factors such as MIF and TGF-β (Zhang et al., 2012).

Pseudotime analysis showed that *TXNRD1* and *LPL* were highly expressed in the early stages of macrophage differentiation, suggesting that they may influence macrophage function through metabolic regulation. As an antioxidant enzyme, *TXNRD1* maintains the pro-inflammatory phenotype of macrophages by scavenging ROS (Kudin et al., 2017), but its overexpression may also induce T cell exhaustion by upregulating PD-L1 (Yang et al., 2024; Budimir et al., 2022). In contrast, *LPL* enhances antigen-presenting capacity by promoting lipid catabolism (Nakamura et al., 2021), and its specific mechanism in LUAD requires further validation.

## 5 Limitations

This study has several important limitations that warrant consideration. First, our single-cell RNA-seq analysis was based on a relatively small cohort (GSE131907), which may restrict the generalizability of our findings regarding the full spectrum of cellular heterogeneity within LUAD. In an effort to bridge this gap, we attempted to validate the prognostic potential of cell-type-specific marker genes in the large, independent TCGA-LUAD bulk RNA-seq cohort (n = 515). However, consistent with the fundamental resolution differences between single-cell and bulk sequencing technologies, this validation yielded limited success—only one marker gene, FLT3, demonstrated significant prognostic value, while the majority did not pass the significance threshold. This outcome undeLimitations.

This study has several important limitations. First, our single-cell RNA-seq analysis relied on a relatively small cohort (GSE131907, n = 22), which may limit the generalizability of findings regarding cellular heterogeneity in LUAD. Consistent with the known challenges of cross-platform validation, attempts to translate cell-type-specific markers into prognostic signals in the bulk TCGA-LUAD cohort (n = 515) yielded limited success—only FLT3 showed significant association—highlighting the difficulty of capturing fine-grained single-cell signatures in aggregated tissue data.

Second, while our multi-omics and Mendelian randomization (MR) analyses provide strong associative and statistically supported causal evidence, direct functional validation is lacking. We did not perform gain- or loss-of-function experiments to demonstrate that modulating the seven prognostic genes directly affects LUAD phenotypes (e.g., proliferation, ERS activation, or butyrate metabolism). Consequently, the proposed mechanistic links remain inferential.

Third, the MR analysis was primarily based on GWAS data from European-ancestry populations, which may limit the generalizability of causal inferences to East Asian LUAD patients, who often exhibit distinct genetic and etiological profiles.

Fourth, although bioinformatic analyses consistently link our signature genes to both ERS and butyrate metabolism pathways, the direct molecular crosstalk between these two processes has not been experimentally tested. Whether butyrate modulates ERS (e.g., via HDAC inhibition) or *vice versa* remains unknown.

Fifth, all predicted drug–target interactions (e.g., furazolidone, flavin) are based solely on computational docking and database scoring. Their actual binding affinity, on-target effects, and anti-tumor activity in LUAD cells have not been validated experimentally.

Finally, despite successful retrospective validation in an independent cohort (GSE31210), the absence of prospective, multi-center clinical validation remains the primary barrier to clinical translation of our prognostic model.

## 6 Conclusion

This study systematically elucidated the key roles of the endoplasmic reticulum stress (ERS) and butyrate metabolism (BM) regulatory networks in LUAD through integrated multi-omics analysis. We successfully constructed a novel molecular marker model with demonstrable clinical predictive value. Critically, by leveraging single-cell resolution, we pinpointed macrophages as a pivotal cellular hub intricately linked to both ERS and BM pathways—a core finding that provides crucial cellular context for interpreting our bioinformatics and MR results.

Our conclusions are further strengthened by a multi-dimensional evidence chain that integrates single-cell trajectory inference, *in vitro* perturbation experiments, and tissue-level protein validation using independent clinical datasets from the Human Protein Atlas (HPA).

In summary, this work represents a preclinical, retrospective investigation that provides a foundational framework for future research. Future studies must prioritize a prospective, multi-center validation trial to rigorously assess the model’s real-world clinical utility. Concurrently, larger single-cell cohorts, spatial genomics, and targeted functional experiments are needed to refine our cellular blueprints and elucidate the precise molecular mechanisms, ultimately bridging the gap between these computational discoveries and their application in precision oncology for LUAD.

## Data Availability

The datasets ANALYZED for this study can be found in the [The Cancer Genome Atlas (TCGA) database] [https://portal.gdc.cancer.gov], and [Gene Expression Omnibus (GEO) database] [http://www.ncbi.nlm.nih.gov/geo/].

## Supplementary material

SUPPLEMENTARY FIGURE S1 SNPs scatter plot.

SUPPLEMENTARY FIGURE S2 Prognostic Gene Mendelian Randomization Analysis with leave - one - out.

SUPPLEMENTARY FIGURE S3 Scatter plot of Mendelian randomization analysis of prognostic genes.

SUPPLEMENTARY FIGURE S4 Correlation analysis of prognostic genes and LUAD.

SUPPLEMENTARY FIGURE S5 Construction and evaluation of risk models.

SUPPLEMENTARY FIGURE S6 Prognostic gene survival curve.

SUPPLEMENTARY FIGURE S7 Single-cell analysis. **(A)** Before the quality control of single-cell data. **(B)** after the quality control of single-cell data. The horizontal coordinate represents single-cell samples, and the vertical coordinates from left to right are: nFeature_RNA, nCount_RNA, percent_mt in sequence. **(C)** Jackstraw diagram. **(D)** Gravel diagram. **(E)** Distribution map of cell types grouped by disease status, where cells of different colors represent different types of cells, and the larger the proportion, the greater the number of cells.

SUPPLEMENTARY FIGURE S8 GSEA enrichment analysis.

SUPPLEMENTARY FIGURE S9 Bubble chart before cell cluster annotation.

SUPPLEMENTARY FIGURE S10 Lung adenocarcinoma – Relative protein expression levels.

SUPPLEMENTARY FIGURE S11 Bubble chart before cell cluster annotation.

SUPPLEMENTARY TABLE S1 Table of differential gene information.

SUPPLEMENTARY TABLE S2 Information table related to prognostic genes.

SUPPLEMENTARY TABLE S3 Information table of GO enrichment analysis of prognostic genes.

SUPPLEMENTARY TABLE S4 Information table of KEGG enrichment analysis of prognostic genes.

SUPPLEMENTARY TABLE S5 Mendelian randomization analysis of prognostic genes for forest plots.

SUPPLEMENTARY TABLE S6 The heterogeneity tests.

SUPPLEMENTARY TABLE S7 The pleiotropy test information table.

SUPPLEMENTARY TABLE S8 Steiger directivity analysis.

SUPPLEMENTARY TABLE S9 Prognostic gene univariate Cox.

SUPPLEMENTARY TABLE S10 Prognostic gene multivariate Cox.

SUPPLEMENTARY TABLE S11 KEGG enrichment analysis of differentially expressed genes in macrophages.

SUPPLEMENTARY TABLE S12 Marker gene table of cell cluster.

SUPPLEMENTARY TABLE S13 GO enrichment analysis of differentially expressed genes in macrophages.

SUPPLEMENTARY TABLE S14 KEGG enrichment analysis of differentially expressed genes in macrophages.

Supplementary Table 15 Multivariate Cox regression table.

## Glossary

LUAD: Lung adenocarcinoma
BM: Butyrate metabolism
WGCNA: Weighted gene co-expression network analysis
GSEA: Gene set enrichment analysis
LRG: Low risk group
HRG: High risk group
ERS: Endoplasmic reticulum stress
UPR: Unfolded protein response
TME: Tumor microenvironment
TCGA: The cancer genome atlas
OS: Overall survival
GEO: Gene expression omnibus
BM-RGs: Butyrate-metabolism-related genes
GSVA: Gene set variation analysis
FC: Fold change
GO: Gene ontology
BP: Biological processes
MF: Molecular functions
KEGG: Kyoto encyclopedia of genes and genomes
STRING: Search tool for the retrieval of interacting genes/proteins
IVW: Inverse variance weighted
MR: Mendelian randomization
HPA: Human protein atlas
LEG: Low-expression group
GSEA: Gene set enrichment analysis
TMB: Tumor mutation burden
UMAP: Uniform manifold approximation and projection
PPI: Protein-protein interaction
RT-qPCR: Reverse transcription quantitative polymerase chain reaction
