# Supplementary figures and images for: Combined single-cell transcriptome and Mendelian randomization to identify and validate prognostic genes associated with endoplasmic reticulum stress and butyrate metabolism in lung adenocarcinoma
Source: Front Genet. 2026 Mar 26;17:1781852. doi: 10.3389/fgene.2026.1781852 (PMC13061388; doi:10.3389/fgene.2026.1781852)

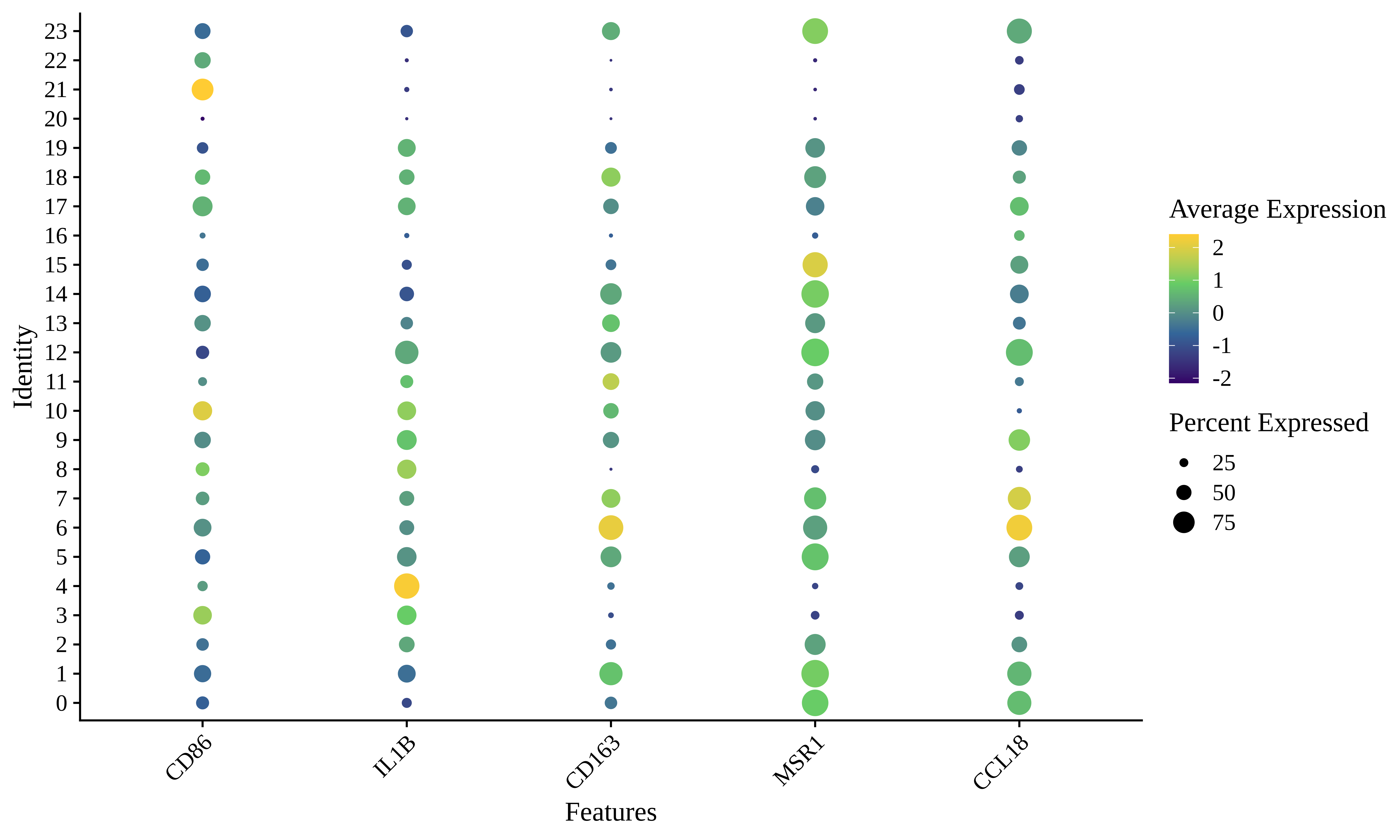

Supplement: Supplementary file 3 [file Image11.png]

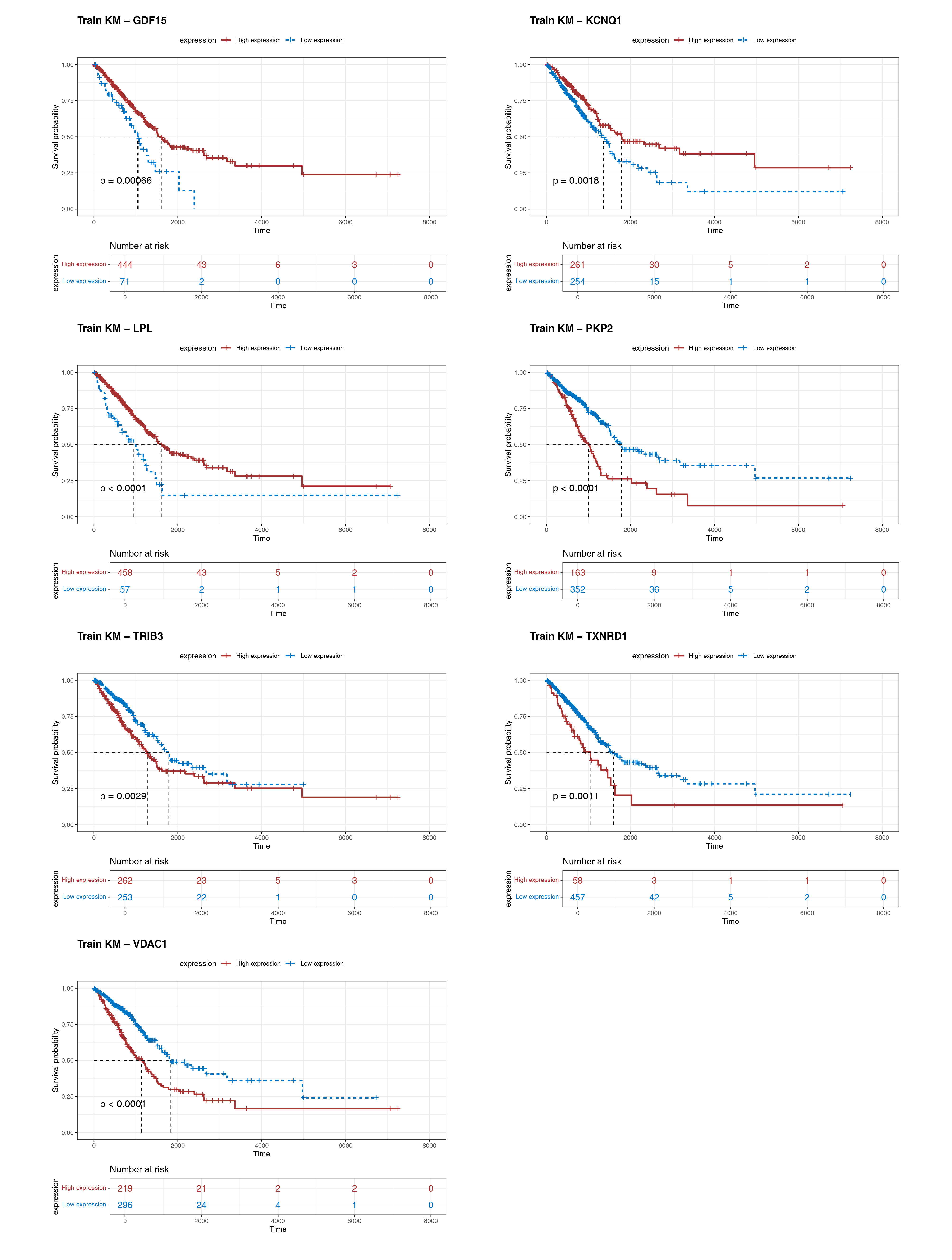

Supplement: Supplementary file 5 [file Image6.tif]

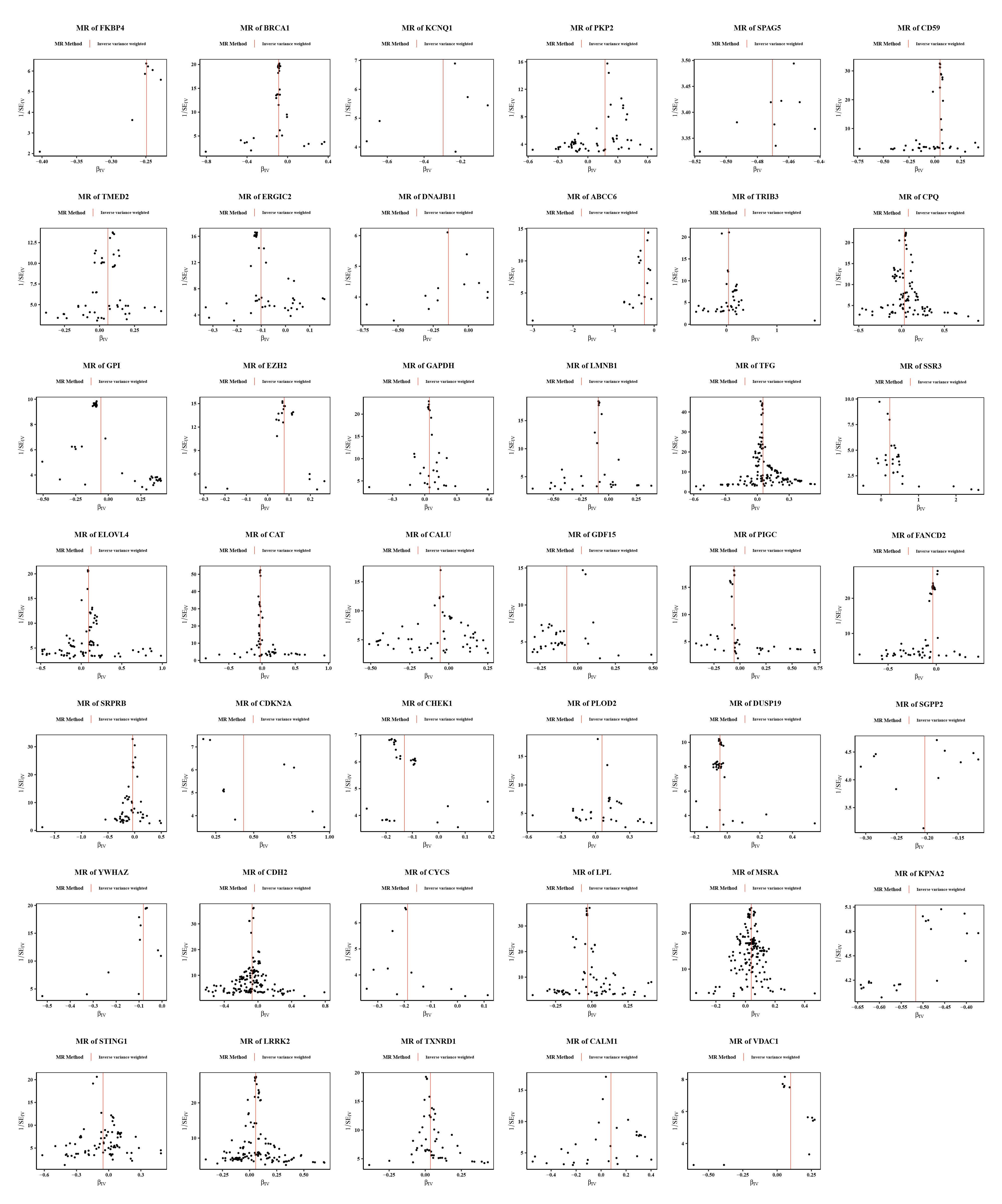

Supplement: Supplementary file 7 [file Image3.tif]

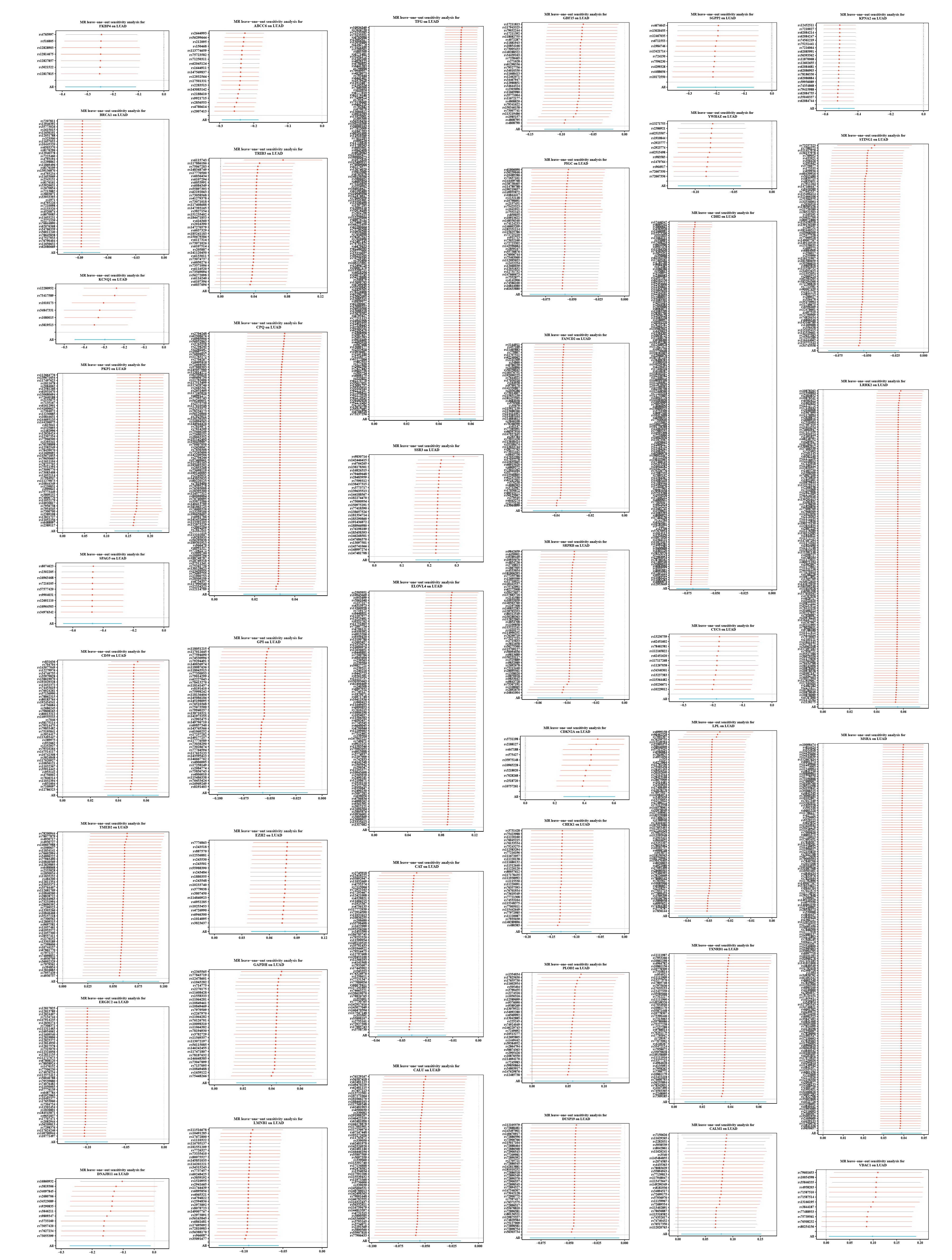

Supplement: Supplementary file 8 [file Image4.tif]

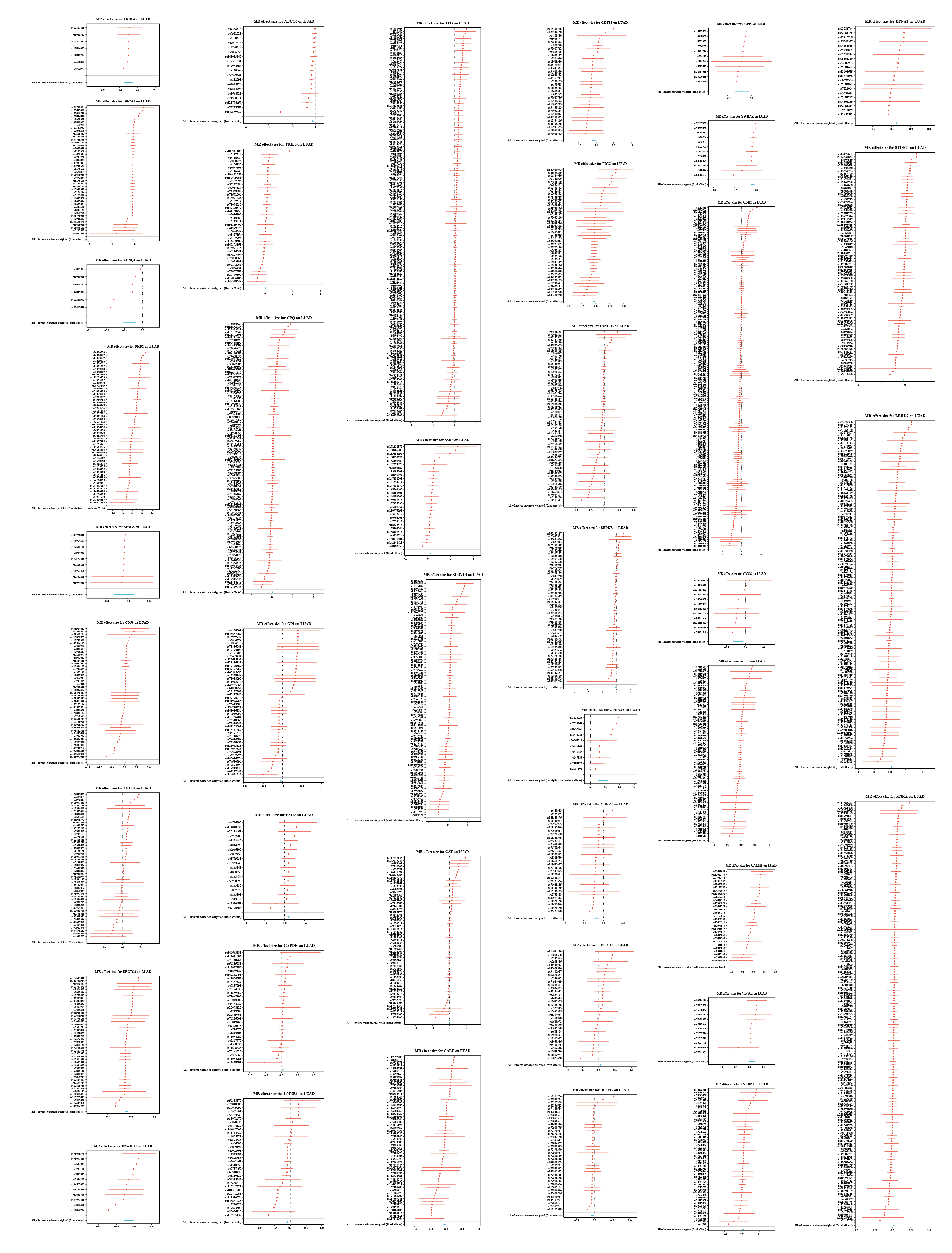

Supplement: Supplementary file 9 [file Image2.tif]

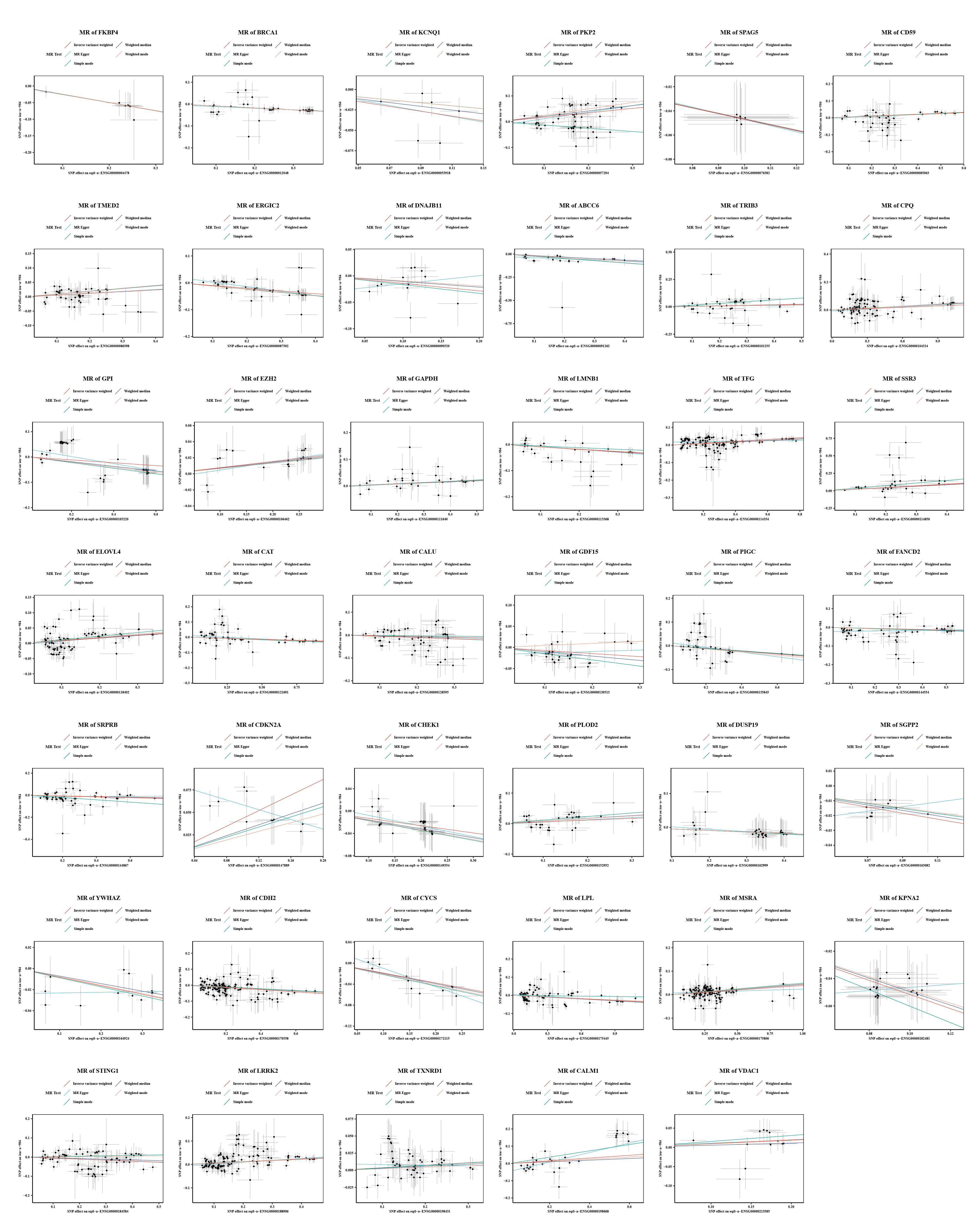

Supplement: Supplementary file 11 [file Image1.tif]

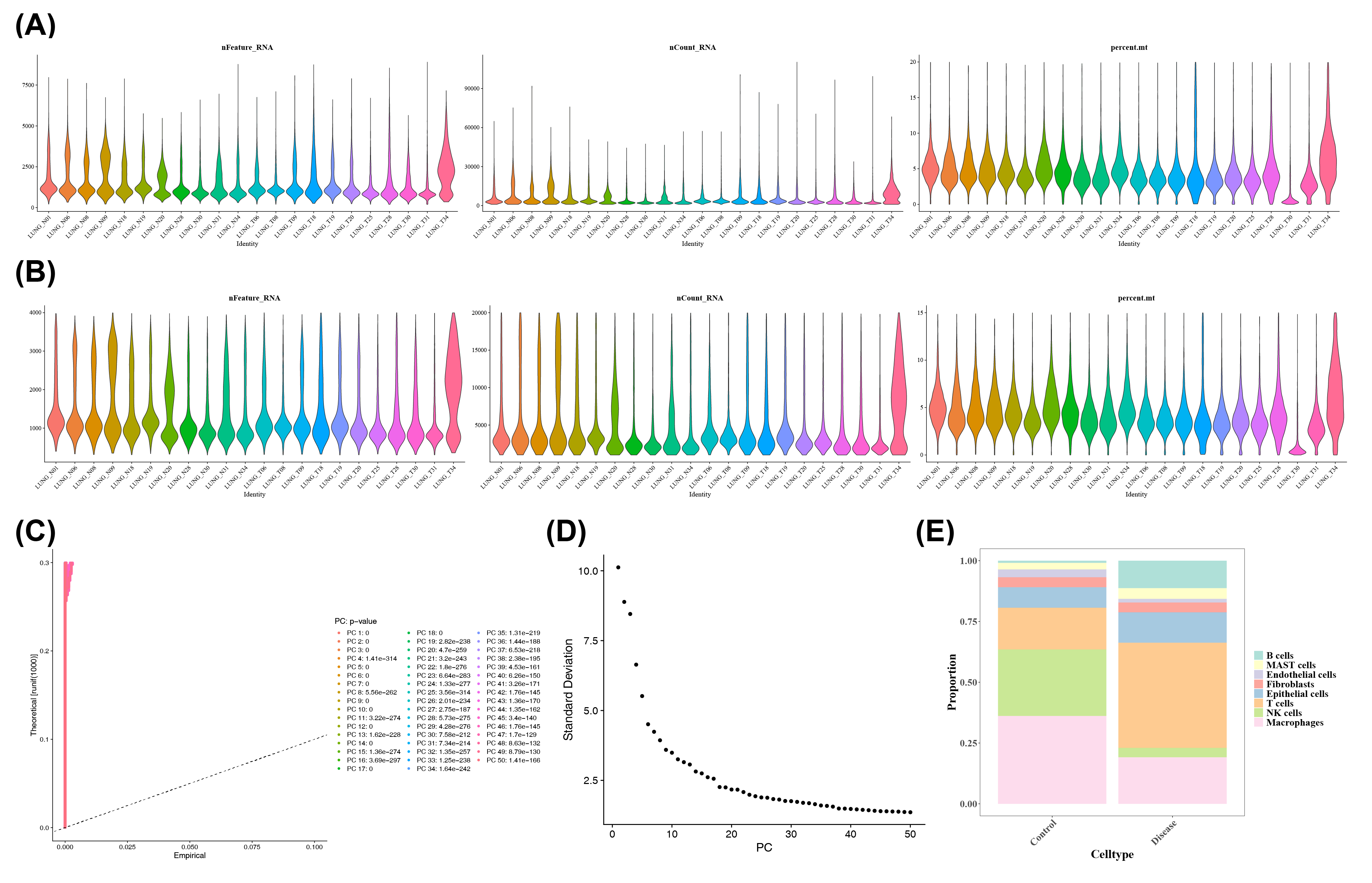

Supplement: Supplementary file 12 [file Image7.tif]

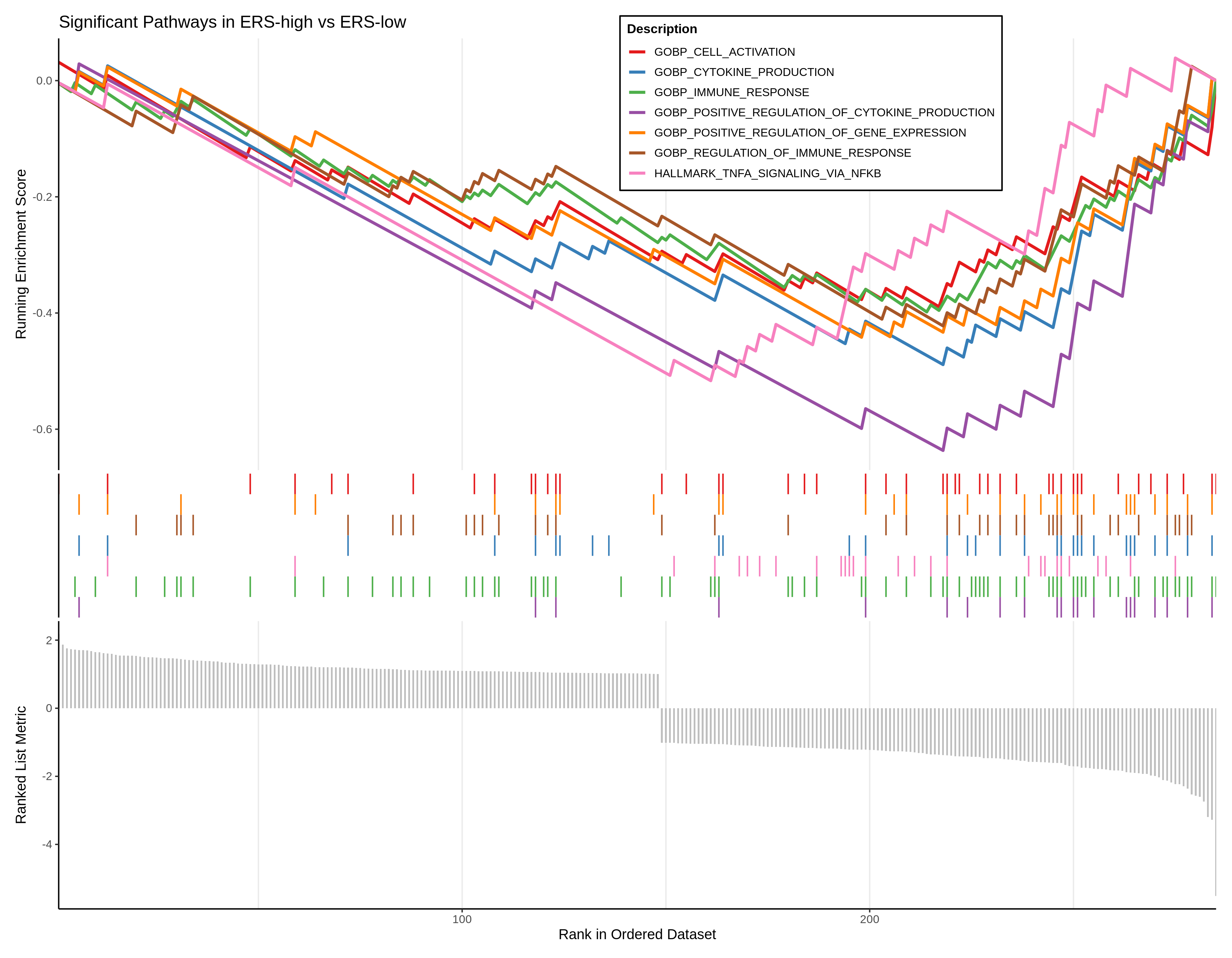

Supplement: Supplementary file 17 [file Image8.png]

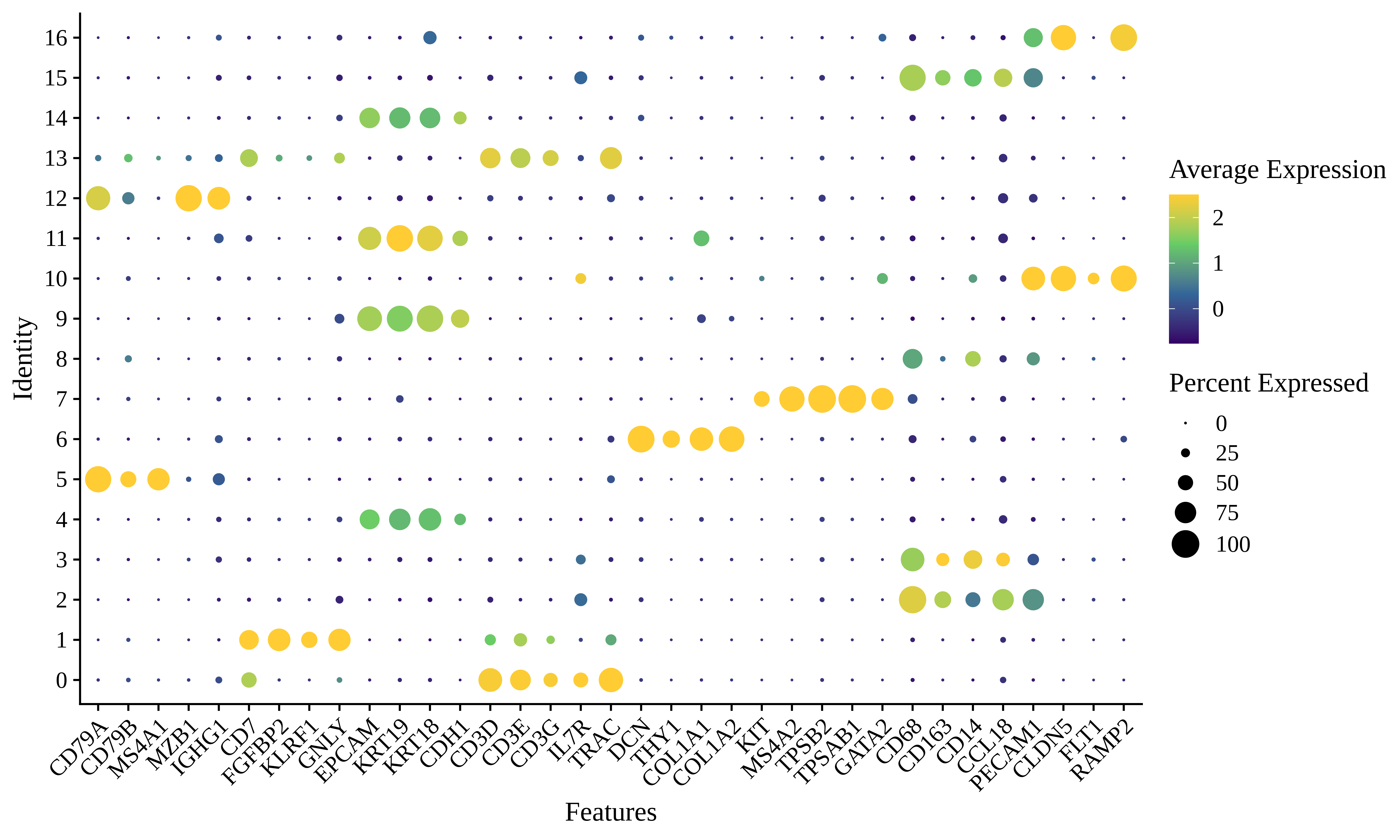

Supplement: Supplementary file 18 [file Image9.png]

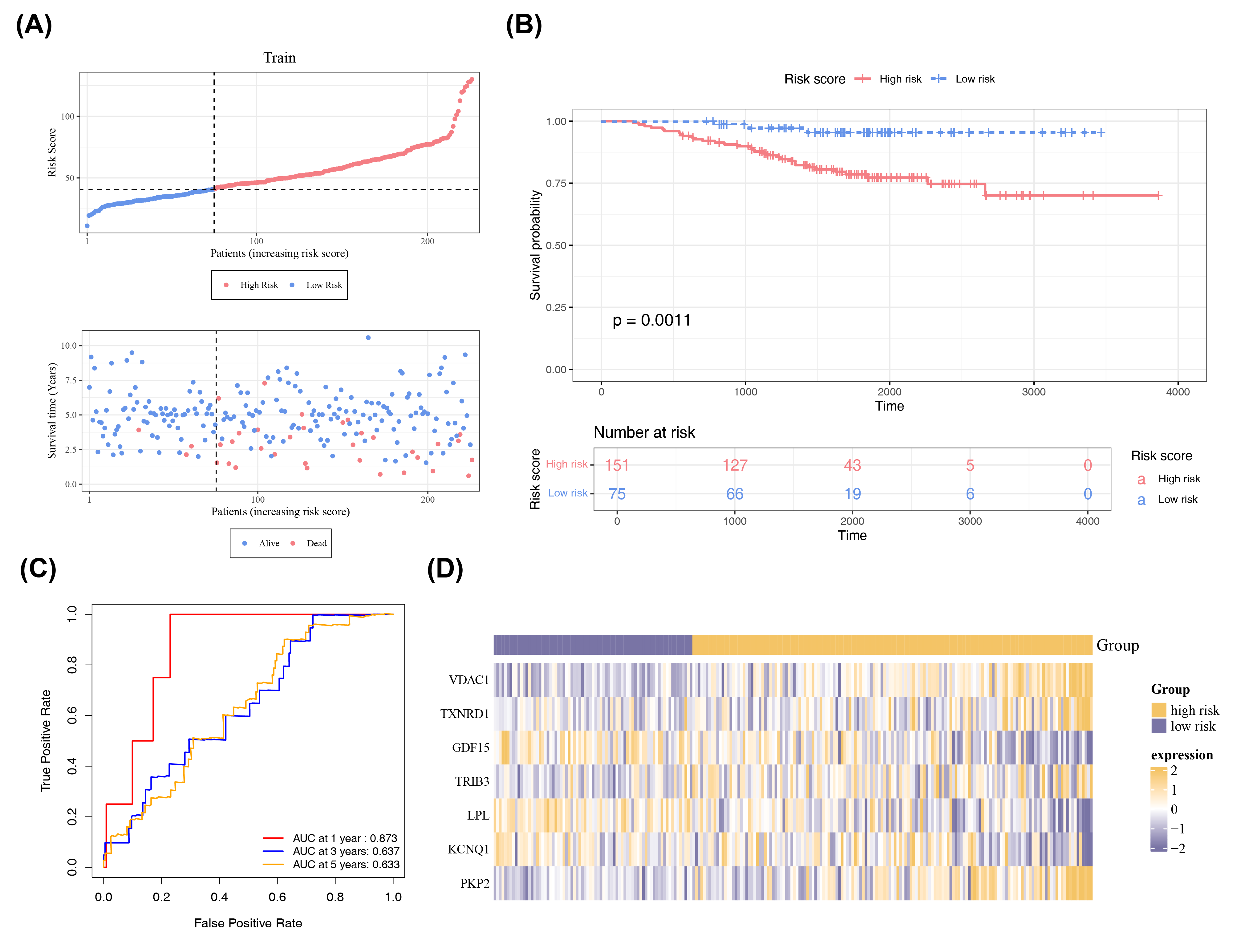

Supplement: Supplementary file 21 [file Image5.tif]

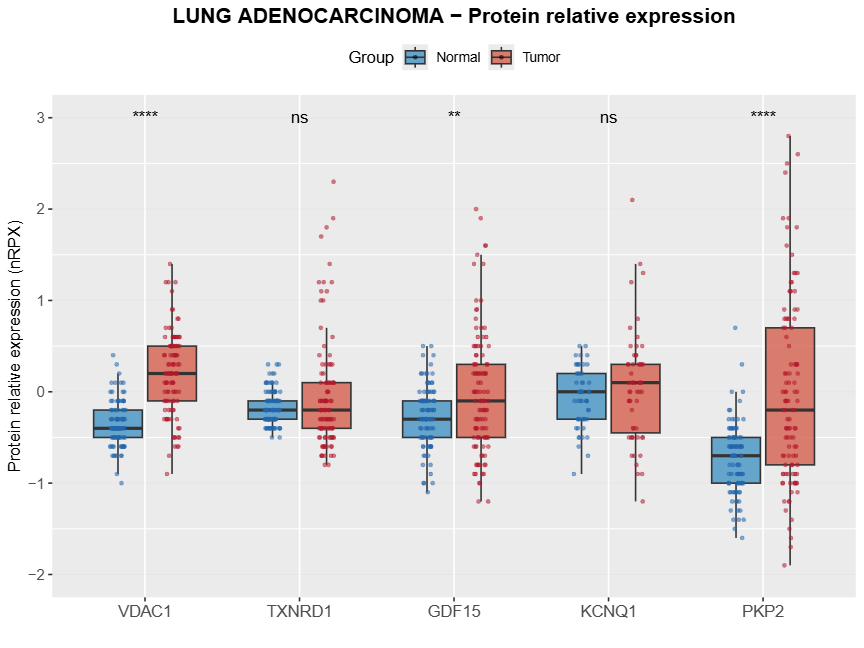

Supplement: Supplementary file 26 [file Image10.png]
